# Diversity of Aromatic Aldehyde Dehydrogenases in *Ceriporiopsis subvermispora*: Insights into Fungal Vanillin Metabolism

**DOI:** 10.1007/s12010-025-05456-1

**Published:** 2025-11-11

**Authors:** Junseok Lee, Takahito Watanabe, Naoko Kobayashi, Ayako Kido, Takashi Watanabe

**Affiliations:** Laboratory of Biomass Conversion, Research Institute for Sustainable Humanosphere, Kyoto University, Gokasho Uji, Kyoto, 611-0011 Japan

**Keywords:** *Ceriporiopsis subvermispora*, Aromatic aldehyde dehydrogenase, Vanillin, Syringaldehyde, *Rhodococcus*, White-rot fungus, Lignin degradation

## Abstract

Lignin-derived aromatic aldehydes are key intermediates in lignocellulosic biomass degradation but can exert cytotoxic effects on microorganisms. *Ceriporiopsis subvermispora* exhibited enhanced glucose consumption and mycelial growth when cultured in the presence of vanillin, suggesting its ability to metabolize lignin-derived aldehydes. We identified 16 putative aldehyde dehydrogenase genes (*Cs*-*aldhA–P*) from *C. subvermispora*, based on sequence homology to known aromatic aldehyde dehydrogenases (*Pc-*ALDH1 and *Pc*-ALDH2) from *Phanerochaete chrysosporium*. These *Cs*-Aldhs shared 65.7–89.7% amino acid identity with *Pc*-ALDHs and contained conserved functional motifs. They were grouped into six clades based on phylogenesis and AlphaFold2-based structural analyses, with pronounced divergence in the architecture of their substrate-binding pockets. Fourteen of these genes were successfully expressed in *Rhodococcus erythropolis*, and their enzymatic activities were evaluated using fluorescence-based NADH assays. *Cs*-AldhJ showed broad specificity and high activity (1.86 ± 0.34 to 5.22 ± 0.05 U/mg), whereas *Cs*-AldhA and *Cs*-AldhK exhibited lower activities, ranging from 0.02 ± 0.00 to 0.08 ± 0.01 U/mg and 0.05 ± 0.01 to 0.38 ± 0.01 U/mg, respectively. The corresponding oxidation products were confirmed by GC–MS, and kinetic analyses revealed mechanistic diversity among the enzymes. These findings highlight the functional diversification of the *Cs*-Aldh family and provide molecular insights into the cellular responses of *C. subvermispora* to lignin-derived aromatic compounds.

**Supplementary Information:**

The online version contains supplementary material available at 10.1007/s12010-025-05456-1.

## Introduction

Lignocellulosic biomass—composed of cellulose, hemicellulose, lignin, and extractives—is the most abundant component of plant cell walls and a major renewable carbon source on Earth. Among these components, lignin is a heterogeneous aromatic polymer accounting for 20–30% of the plant cell wall. It forms lignin-carbohydrate complexes (LCCs) through covalent and hydrogen bonding with hemicellulose and associates with cellulose microfibrils, contributing to the structural rigidity and recalcitrance of the cell wall to microbial and chemical degradation [1–3]. In natural environments, lignin is primarily depolymerized by radical-mediated reactions catalyzed by peroxidases secreted by wood-decaying fungi in the phylum Basidiomycota, resulting in a variety of aromatic compounds [4]. These insights have spurred intensive research on lignin depolymerization to facilitate cellulose separation and the production of reducing sugars via enzymatic saccharification. The resulting lignin-derived aromatic compounds are further metabolized by bacteria and other microorganisms through enzymatic pathways, ultimately leading to mineralization [5].

Recent genomic studies of wood-decaying fungi have challenged the classical dichotomy between “white-rot” and “brown-rot” fungi [6]. Many fungi traditionally classified as white-rot are recognized for their ability to completely degrade lignin in wood. However, despite this ligninolytic capacity, many white-rot fungi degrade low-molecular-weight aromatic compounds, such as vanillin and syringaldehyde, only slowly. At concentrations of approximately 1–10 mM, these compounds inhibit fungal growth and enzyme production in a concentration-dependent manner [7–13].

Aldehyde dehydrogenases (Aldhs) are a conserved enzyme superfamily found in all domains of life, from prokaryotes such as bacteria to eukaryotes including plants, animals, and humans. These enzymes catalyze the NAD(P)^+^-dependent oxidation of toxic aldehydes to their corresponding carboxylic acids, thereby playing key roles in intracellular detoxification, oxidative stress responses, metabolic regulation, and development. Although the functions of Aldhs in human health are well studied, their diversity and roles in fungi remain largely underexplored [14].

Aromatic aldehyde-degrading Aldhs have been characterized in bacteria such as *Sphingobium* sp. SYK-6 [5]. Although biochemical studies on lignin depolymerization are extensive, relatively little is known about how fungi metabolize lignin-derived aromatic aldehydes such as vanillin. In the white-rot fungus *Phanerochaete chrysosporium*, exposure to vanillin has been shown to enhance glucose consumption, inhibit mycelial growth, and shift carbon flux from the glyoxylate cycle to the TCA cycle [15]. To date, only two aromatic Aldhs, *Pc*-ALDH1 and *Pc*-ALDH2, have been isolated from *P. chrysosporium* and characterized for their roles in vanillin catabolism [16]. Comprehensive analyses of fungal Aldhs in other white-rot fungi, however, remain lacking.

To further investigate the detoxification and metabolism of aromatic aldehydes in white-rot fungi, we focused on the selective lignin degrader *Ceriporiopsis subvermispora*. Although genomes of several white-rot fungi (e.g., *P. chrysosporium*, *Pleurotus ostreatus*) have been sequenced, *C. subvermispora* represents a unique model due to its selective lignin degradation—preferential lignin removal with minimal cellulose damage—and its production of ceriporic acids, natural chelators that suppress the cellulolytic Fenton reaction and enhance manganese peroxidase (MnP)-mediated ligninolysis [17, 18]. Elucidating the diversity of Aldhs in this fungus is therefore essential for understanding how white-rot fungi adapt to cytotoxic aromatic aldehydes during ligninolysis. Its selective ligninolytic activity also highlights its potential in biopulping and as a biological pretreatment agent for enzymatic saccharification and fermentation of woody biomass [19–21]. In this study, we tested the hypothesis that *C. subvermispora* encodes a functionally diversified family of aromatic aldehyde dehydrogenases (*Cs*-Aldhs), in which structural variation underlies substrate selectivity and kinetic specialization toward lignin-derived aldehydes. To address this, we identified and characterized 16 *Cs*-Aldhs using genomic, phylogenetic, structural, and biochemical approaches, thereby providing molecular insight into fungal adaptation to these cytotoxic aromatic compounds.

## Materials and Methods

### Fungal and Bacterial Strains and their Culture Conditions

*Ceriporiopsis subvermispora* ATCC 90467 was obtained from the American Type Culture Collection. Unless otherwise noted (e.g., *C. subvermispora* strain B), all references to *C. subvermispora* in this study indicate strain ATCC 90467. The fungus was maintained on potato dextrose agar (PDA; Shimadzu Diagnostics Corporation, Tokyo, Japan) and cultured in a complete synthetic medium, BIII liquid medium, at 28 °C, as described previously [22, 23] (Table S1). *P. chrysosporium* ATCC 34541 was cultivated under the same conditions.

A 7-mm-diameter mycelial plug from a fully colonized PDA plate was transferred to fresh PDA and incubated at 28 °C for 7 days in the dark. The resulting mycelium was scraped into 2 mL of BIII medium, and 1 mL of this suspension was inoculated into 49 mL of BIII medium in a 300-mL Erlenmeyer flask for pre-cultivation at 28 °C. After 7 days, 1 mL of the pre-culture was transferred to 48 mL of fresh BIII liquid medium in a new 300-mL Erlenmeyer flask and incubated statically for another 7 days. For vanillin treatment, 1 mL of a 50 mM vanillin solution or BIII medium (control) was then added, followed by a further 7-day incubation. Vanillin was used at a final concentration of 2 mM, a level commonly reported to inhibit fungal growth in other white-rot fungi [7–13]. However, preliminary tests confirmed that this concentration did not inhibit the growth of *C. subvermispora* ATCC 90467, allowing us to examine its physiological and metabolic responses under conditions inhibitory to many related species. Mycelia from both control and vanillin-treated cultures were harvested for subsequent analyses.

*Escherichia coli* JM109 (Toyobo Co. Ltd., Osaka, Japan) and *Rhodococcus erythropolis* L88 (Hokkaido System Science Co., Ltd., Sapporo, Japan) were used for gene cloning and heterologous protein expression, respectively. *E. coli* was grown on Luria-Bertani (LB) agar containing 50 µg/mL carbenicillin at 37 °C, while *R. erythropolis* was cultured on LB agar containing 10 µg/mL tetracycline at 28 °C.

### Measurement of Glucose Consumption and Preparation of Dried Mycelia

Culture supernatants of *C. subvermispor*a grown in BIII liquid medium with or without vanillin were filtered through Miracloth (25 μm pore size; Merck Millipore, Darmstadt, Germany) as previously described [22]. Glucose concentrations were determined colorimetrically using the Glucose CII Test Wako (FUJIFILM Wako Pure Chemical Corporation, Osaka, Japan) after heat treatment at 100 °C for 10 min, following the manufacturer’s protocol.

To remove the extracellular polysaccharide sheath formed in response to vanillin exposure, mycelia were washed twice with 50% ethanol, as described by Watanabe et al. [23]. The mycelial pellets were then blotted dry between paper towels, followed by dehydration at 50 °C for 1 h and subsequent drying at 70 °C overnight.

### DNA Manipulation, Sequencing, and RNA Preparation

DNA manipulations were performed as described by Sambrook et al. [24]. Plasmid DNA was extracted using the ChargeSwitch-Pro Plasmid Miniprep Kit (Life Technologies, Carlsbad, CA, USA) and cloned into the pBluescript II KS (+) vector (Toyobo Co. Ltd.). DNA sequencing was performed by the Uji DNA Sequence Core at Kyoto University.

Total RNA was extracted from undried *C. subvermispora* pellets using ISOGEN II solution (Nippon Gene Co. Ltd., Tokyo, Japan) and a Polytron PT1300D homogenizer (Kinematica AG, Malters, Switzerland). RNA was precipitated with 2-propanol and washed twice with 80% ethanol. The resulting pellet was air-dried, dissolved in nuclease-free water, and stored at − 80 °C until use.

### PCR

All primers used in this study were synthesized by FASMAC Co., Ltd., Atsugi, Japan (Table S2). For colony PCR, 2×GoTaq Green Master Mix (Promega, Madison, WI, USA) was used to confirm whether the Aldh genes identified in *C. subvermispora* strain B were also present in strain ATCC 90467 and to verify their orientation relative to the *tipA* promoter of the pTipQT vectors for *R. erythropolis* L88 (Fig. S2). PCR was performed for 30 cycles under the following conditions: denaturation at 98 °C for 10 s, annealing at 55 °C for 15 s, and extension at 68 °C for 1.5 min.

### Phylogenetic Analysis

Sixteen *Cs*-Aldh amino acid sequences from *C. subvermispora* ATCC 90467 were aligned using ClustalW [25], and a neighbor-joining phylogenetic tree was constructed using MEGA 10.1.6 [26]. Bootstrap analysis was conducted with 1,000 replicates. Twelve representative homologs were selected from BLASTP search results using *Cs*-Aldh sequences as queries, encompassing functionally diverse types such as aromatic, aldehyde dehydrogenases, betaine aldehyde dehydrogenases, succinate-semialdehyde aldehyde dehydrogenases, methylmalonate-semialdehyde aldehyde dehydrogenases, and vanillin dehydrogenases. To investigate broader evolutionary relationships, an expanded phylogenetic tree was generated incorporating Aldh sequences from multiple taxonomic groups, and bootstrap analysis was performed with 10,000 bootstrap replicates (Fig. S5).

### Protein 3D Modeling Analysis

The amino acid sequences of *Cs*-Aldhs were submitted to ColabFold [27–29] to predict their three-dimensional structures, and the five most probable models were generated in Protein Data Bank (PDB) format. These models were visualized using PyMOL (The PyMOL Molecular Graphics System, Version 2.5; Schrödinger, LLC) (Figs. 3A–C and Fig. S6) and compared to human aldehyde dehydrogenase 2 (ALDH2) (UNIPROT: P05091). For detailed comparison of substrate-binding channels, the structure of *Cs*-AldhA was superimposed with those of *Cs*-AldhF and *Cs*-AldhH (Fig. 3B), and distances between selected substrate-interacting residues and the catalytic cysteine (*u–z* in Fig. S6 and Table S4) were measured. Each distance was calculated across five models to obtain average values and standard deviations.

### Construction of Cs-Aldhs Expression Vectors and Transformation of Rhodococcus erythropolis L88

*Cs-*Aldh coding sequences (CDSs) were initially cloned into pBluescript II KS (+) (Toyobo Co. Ltd.) for sequence verification. They were subsequently modified to include six histidine codons—at the N-terminus for *Cs*-AldhF, *Cs*-AldhM, and *Cs*-AldhO, and at the C-terminus for the remaining *Cs*-Aldhs—and flanked with *Nde*I restriction sites. These fragments were inserted into *Rhodococcus* expression vectors (pTipQT1 or pTipQT2, Hokkaido System Sciences Co. Ltd.) under the control of the thiostrepton-inducible *tipA* promoter, and the resulting constructs were verified by sequencing (Fig. S2). The expression plasmids (200 ng) were electroporated into *R. erythropolis* L88 competent cells (Hokkaido System Science Co., Ltd.) using a GENE PULSER II electroporator (Bio-Rad Laboratories, Inc., Hercules, CA) according to the manufacturer’s instructions (1.6 kV, 25 µF, 400 Ω for a 1-cm cuvette). Transformants were selected on LB agar plates containing 10 µg/mL tetracycline and incubated at 28 °C for 3–5 days in the dark.

### Protein Expression, Preparation, and Purification

*R. erythropolis* transformants were pre-cultured in 5 mL of liquid LB medium supplemented with 10 µg/mL tetracycline at 28 °C for 36–48 h with reciprocal shaking at 200 rpm in the dark. The pre-cultures were then transferred into 95 mL of fresh LB medium containing 10 µg/mL tetracycline in a 500-mL baffled Erlenmeyer flask and incubated under the same conditions. After 8 h, thiostrepton (10 µg/mL; LKT Laboratories, Inc., Saint Paul, MN, USA), dissolved in dimethyl sulfoxide, was added to induce *Cs*-Aldh expression, and the cultures were incubated overnight. Cells were harvested by centrifugation at 6,000 × *g* for 10 min at 4 °C and stored at − 80 °C until use.

Cell pellets were resuspended in xTractor Buffer (Takara Bio Inc.) and lysed using an ultrasonic disruptor UD-201 (Tomy Seiko Co., Ltd., Tokyo, Japan) under the following conditions: OUTPUT 3, DUTY 50, TIME 10. After centrifugation at 20,000 × *g* for 15 min at 4 °C, the supernatants were collected and analyzed by SDS-PAGE [30].

For purification, His-tagged *Cs*-Aldhs were bound to TALON metal affinity resin (Takara Bio Inc.), washed, and eluted with imidazole-containing buffer according to the manufacturer’s protocol. The eluted proteins were dialyzed against MN Buffer (20 mM MOPS, 150 mM NaCl, pH 7.4) for 24 h at 4 °C using a D-Tube Dialyzer Maxi (MWCO 12–14 kDa, Merck KGaA, Darmstadt, Germany), and stored at − 80 °C until use in an enzyme assay. Protein concentrations were determined using the 2D Quant Kit (Cytiva, Marlborough, MA).

### NADH Fluorescence-based Enzyme Activity Assay

*Cs*-Aldh activity was measured using a fluorescence-based assay that detects NADH production, with excitation at 340 nm and emission at 450 nm (integration time:20 µs). Each reaction (100 µL total volume) contained 100 µg of purified enzyme, 50 mM potassium phosphate buffer (pH 8.0), 0.5 mM NAD⁺, and 0.5 mM of an aromatic aldehyde substrate (vanillin, syringaldehyde, protocatechualdehyde, or *p*-hydroxybenzaldehyde). Fluorescence was monitored over a 20 min period at 25 °C using an Infinite M200 plate reader (Tecan Group Ltd., Männedorf, Switzerland). Background fluorescence was subtracted using reactions without enzyme. NADH concentrations were calculated from a standard curve (Fig. S1B), and specific activity was expressed as µmol of NADH produced per minute per milligram protein.

### GC–MS Analysis of Aromatic Acids Produced from Aromatic Aldehydes by Cs-Aldhs

Aromatic acids produced in *Cs*-Aldh-catalyzed reactions were identified by gas chromatography–mass spectrometry (GC–MS). Each reaction (1 mL) contained 100 µg of purified *Cs*-Aldh, 50 mM potassium phosphate buffer (pH 8.0), 0.5 mM NAD⁺, and 0.5 mM of an aromatic aldehyde substrate (vanillin, syringaldehyde, protocatechualdehyde, or *p*-hydroxybenzaldehyde). Reactions were incubated at 28 °C in the dark for 2 h. After ethyl acetate extraction and derivatization with MSTFA + 1% TMCS (Thermo Fisher Scientific, Waltham, MA, USA), samples were analyzed using a DB-5MS column operated in electron ionization (EI) mode. Chromatographic conditions followed a previously described protocol [31].

### Nucleotide Accession Numbers

The nucleotide sequences reported in this study have been submitted to the DDBJ/ENA/GenBank databases under accession numbers LC662176–LC662191.

## Results

### Glucose Consumption and Fungal Growth of C. subvermispora in Response to Vanillin Exposure

The addition of exogenous vanillin has previously been reported to enhance glucose consumption while inhibiting fungal growth in *P. chrysosporium* [16]. To examine whether vanillin can support fungal growth as a sole carbon source, *C. subvermispora* was incubated at 28 °C for 7 days in medium supplemented with 2 mM vanillin but lacking glucose. No visible growth was observed, indicating that vanillin alone is not sufficient to sustain its growth (Fig. S4A).

**Fig. 1:**
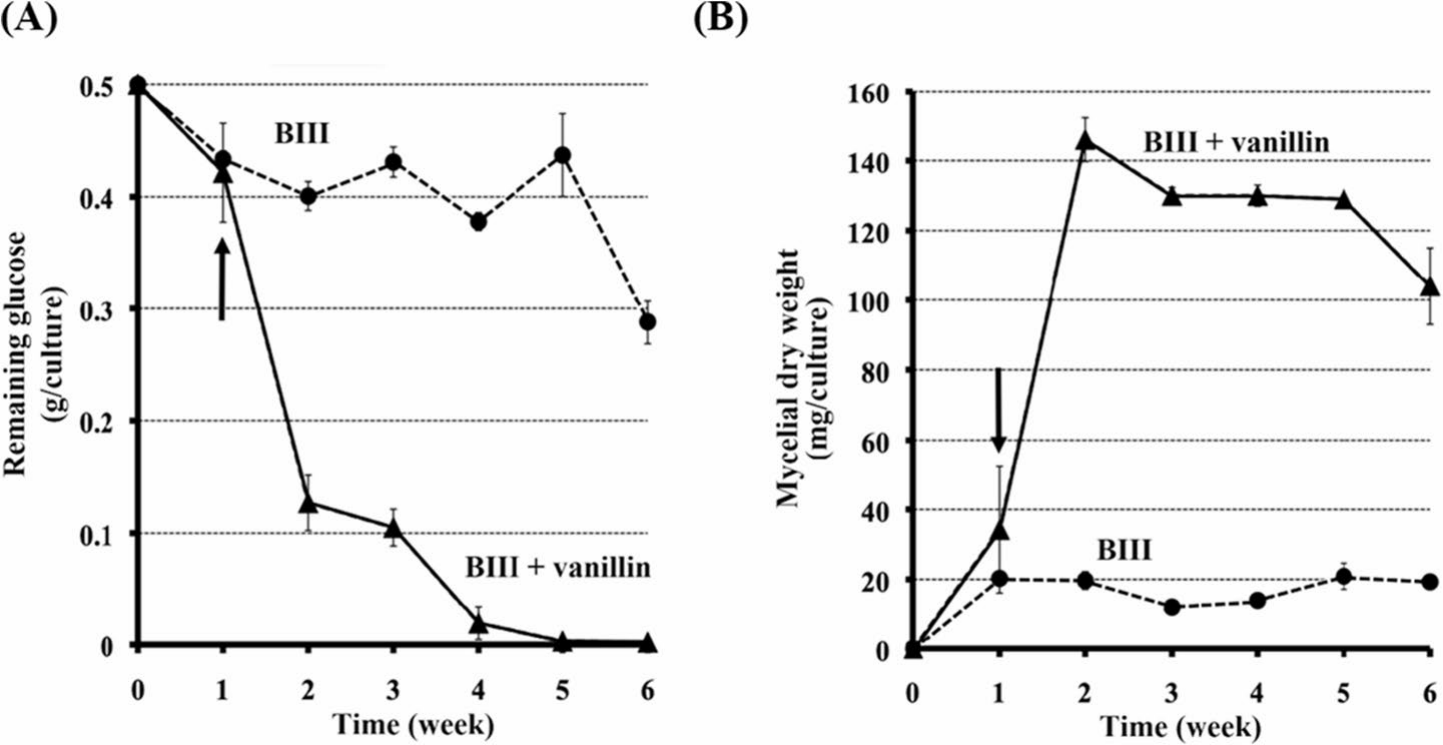
Glucose consumption and mycelial growth of *C. subvermispora* in response to vanillin. Time courses of glucose consumption **(A)** and mycelial dry weight **(B)** during the growth of *C. subvermispor*a ATCC 90467 in BIII medium, a fully defined synthetic liquid medium, in the absence (circles) or presence (triangles) of vanillin. The black arrow indicates the time point at which 2 mM vanillin was added to the medium

When vanillin was added to glucose-containing BIII medium, *C. subvermispora* ATCC 90467 exhibited markedly enhanced glucose consumption (Fig. 1A) and nearly a sevenfold increase in mycelial dry weight compared to the control (Fig. 1B). In contrast, *P. chrysosporium* showed growth inhibition under the same conditions. Consistent with these findings, GC–MS analysis confirmed that vanillin was degraded in *C. subvermispora* cultures, whereas it remained detectable in *P. chrysosporium* cultures (Figs. S4B and S4C). To minimize confounding effects of early exposure, vanillin was added after a 7-day pre-incubation in all subsequent experiments.

### Screening and Cloning of Aldh Genes from C. subvermispora Strain ATCC 90467

To identify genes potentially involved in the detoxification of aromatic aldehydes in *C. subvermispora*, a BLAST search was performed using the amino acid sequences of two known aromatic aldehyde dehydrogenases from *P. chrysosporium* (*Pc*-ALDH1 and *Pc*-ALDH2) as queries, against the JGI genome database of *C. subvermispora* strain B (https://mycocosm.jgi.doe.gov/Cersu1/Cersu1.home.html; [32]). Sixteen candidate genes, designated *Cs*-*aldh*A through *Cs-aldhP*, were identified (Table 1). The presence of these genes in strain ATCC 90467 was confirmed by PCR and sequencing. The predicted *Cs*-Aldh proteins shared high amino acid identity (96.16%–99.88%) with those from the strain B sequences and moderate identity (66.1%–89.7%) with *Pc*-ALDHs. All *Cs*-Aldhs possessed conserved motifs characteristic of aldehyde dehydrogenases, including the catalytic residues Glu264 and Cys295 (as in *Cs*-AldhA), as well as a glycine-rich NAD⁺-binding motif (GXGXXXG), as described by Perozich et al. [14, 33; Fig. S3]. Structural variations were observed in motifs 9 and 10 in several *Cs*-Aldhs. Transmembrane domain prediction using SOSUI and TMHMM indicated that seven *Cs*-Aldhs are potentially membrane-associated (Table S3). Among them, *Cs*-AldhA through *Cs*-AldhD showed particularly high sequence identity (> 80%) with *Pc*-ALDH1 and *Pc*-ALDH2 (Table 1), supporting their potential roles in aromatic aldehyde metabolism.

**Table 1:** The 16 aldehyde dehydrogenases from *C. subvermispora* strain ATCC 90467

Protein name^a)^	JGI Annotation (putative function)	Length	pI^b^	MW^c)^	(vs. Pc-ALDH1) Identities^d)^	(vs. *Pc*-ALDH2)
*Cs*-AldhA (139148)	Aldehyde dehydrogenase (NAD^+^)	497 aa	6.08	53.83	83.3%	85.9%
*Cs*-AldhB (78973)	Aldehyde dehydrogenase (NAD^+^)	497 aa	6.17	54.14	81.7%	89.7%
*Cs*-AldhC (120461)	Aldehyde dehydrogenase (NAD^+^)	497 aa	5.49	54.18	81.1%	89.3%
*Cs*-AldhD (80654)	Aldehyde dehydrogenase (NAD^+^)	504 aa	5.51	54.17	81.7%	80.7%
*Cs*-AldhE (139148)	Succinate-semialdehyde dehydrogenase (NAD(P)^+^)	492 aa	5.56	52.05	70.2%	73.8%
*Cs*-AldhF (118059)	Aldehyde dehydrogenase (NAD^+^)	482 aa	5.37	52.39	74.3%	74.2%
*Cs*-AldhG (80579)	Aldehyde dehydrogenase (NAD(P)^+^)	507 aa	5.41	55.28	69.0%	71.1%
*Cs*-AldhH (87324)	Aldehyde dehydrogenase (NAD^+^)	473 aa	6.61	51.43	73.6%	73.5%
*Cs*-AldhI (96047)	Aldehyde dehydrogenase (NAD(P)^+^)	476 aa	5.67	51.45	73.0%	71.9%
*Cs*-AldhJ (116214)	Succinate-semialdehyde dehydrogenase (NAD(P)^+^)	486 aa	5.87	51.83	69.8%	68.8%
*Cs*-AldhK (79758)	Methylmalonate-semialdehyde dehydrogenase (NAD^+^)	525 aa	7.08	56.01	68.0%	70.2%
*Cs*-AldhL (151449)	Benzaldehyde dehydrogenase (NAD^+^)	486 aa	6.11	51.85	65.7%	68.14%
*Cs*-AldhM (96582)	Succinate-semialdehyde dehydrogenase (NAD(P)^+^)	453 aa	6.28	48.56	66.14%	68.3%
*Cs*-AldhN (115060)	Benzaldehyde dehydrogenase (NAD^+^)	486 aa	5.52	51.77	66.5%	67.4%
*Cs*-AldhO (96599)	Benzaldehyde dehydrogenase (NAD^+^)	453 aa	5.44	53.83	69.7%	66.1%
*Cs*-AldhP (110692)	Methylmalonate-semialdehyde dehydrogenase (NAD^+^)	546 aa	6.69	58.39	70.3%	72.0%

a) The numbers in brackets are protein IDs for *C. subvermispora *strain B, available on the JGI website (http://genome.jgi-psf.org/Cersu1/Cersu1.home.html).

b) Theoretical pI values calculated using Serial Cloner 2.6.1 software.

c) Theoretical molecular masses (kDa) calculated using Serial Cloner 2.6.1 software.

d) Protein IDs 2981181 and 137014 in the JGI database of *P. chrysosporium strain* RP-78 (34).

### Phylogenetic Analysis of Cs-Aldhs and Related Enzymes Across Diverse Species

**Fig. 2:**
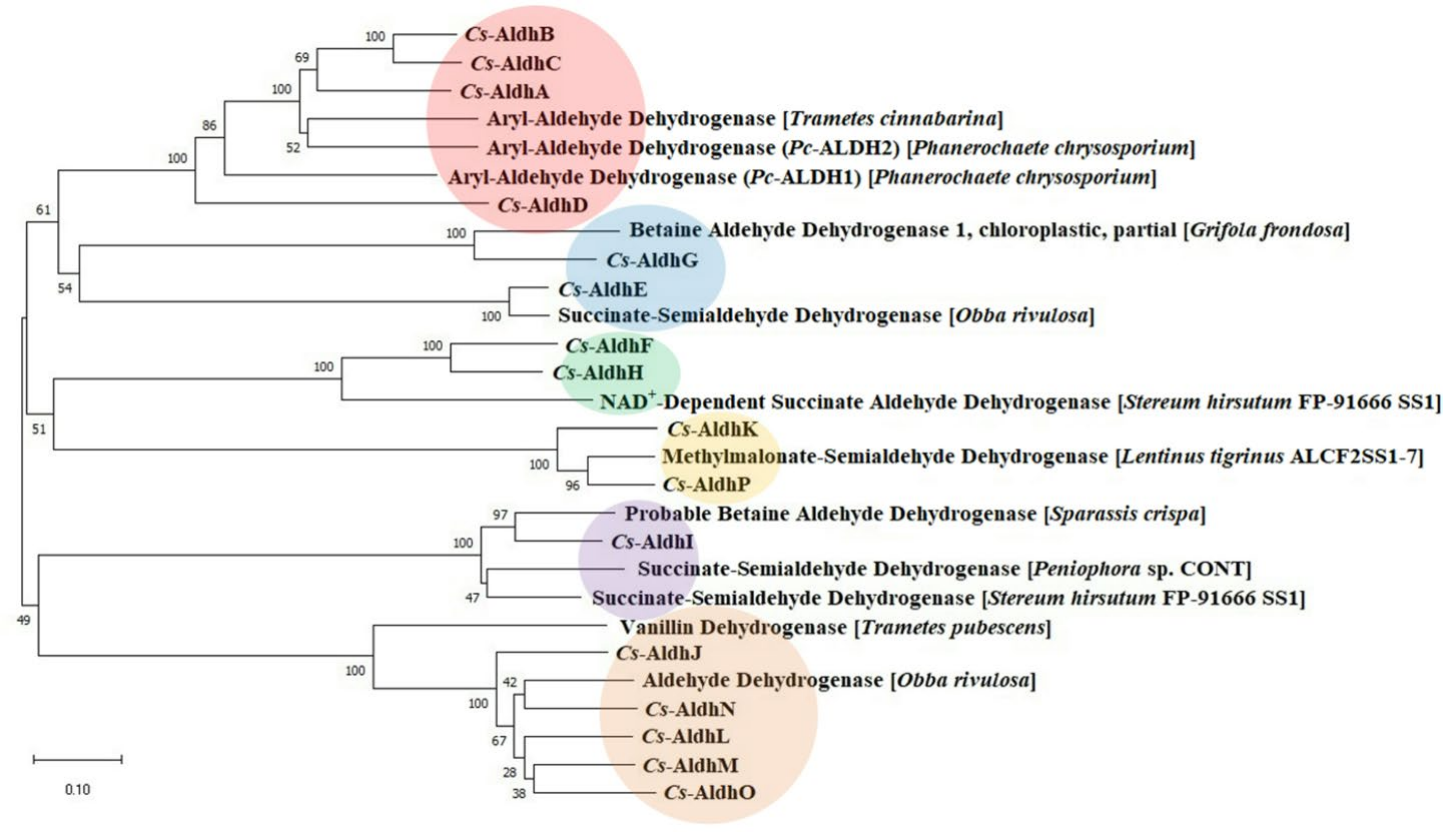
Phylogenetic analysis of 16 *Cs*-Aldhs from *C. subvermispora* ATCC 90467 and homologous enzymes from ligninolytic fungi. Six major clades were identified by neighbor-joining analysis based on amino acid sequences. Twelve representative fungal homologs were selected from approximately 100 BLASTP hits using *Cs*-Aldhs as queries. These include: an aromatic Aldh from *Trametes cinnabarina *(AKS10559.1); two aromatic Aldhs, *Pc*-ALDH1 and *Pc*-ALDH2, from *P. chrysosporium*, reannotated as Protein IDs 2981181 and 137014, respectively, in the updated *P. chrysosporium* RP-78 database (34); two betaine Aldhs from *Grifola frondosa* (OBZ76685.1) and *Sparassis crispa* (XP_027616657.1); three succinate-semialdehyde dehydrogenases from *Obba rivulosa* (OCH95922.1), *Stereum hirsutum* (XP_007298338.1), and *Peniophora* sp. (KZV75500.1); an NAD^+^-dependent succinate Aldh from *S. hirsutum* (XP_007308375.1); a methylmalonate-semialdehyde dehydrogenase from *Lentinus tigrinus* (RPD82059.1); a vanillin dehydrogenase from *T. pubescens* (OJT02400.1); and an Aldh from *O. rivulosa* (OCH86492.1). Accession numbers beginning with two letters (e.g., XP_) refer to NCBI RefSeq entries, while those beginning with three letters (e.g., AKS, OBZ) refer to GenBank entries. Colored circles indicate clade groupings of closely related proteins

To investigate the functional diversity of the identified *Cs*-Aldhs, phylogenetic analysis was performed by comparing aldehyde dehydrogenases (Aldhs) from *C. subvermispora* with homologous enzymes from various fungal species (Fig. 2). The 28 Aldh sequences clustered into six major clades. For example, *Cs*-AldhA–D grouped with aromatic Aldhs from *P. chrysosporium* [16, 34] and *Trametes cinnabarina* (red circle), whereas *Cs*-AldhJ and *Cs*-AldhL–O clustered with a vanillin dehydrogenase from *T. pubescens* (brown circle).

To examine broader evolutionary divergence, an expanded phylogenetic tree was constructed including representative Aldhs from bacteria, Ascomycota fungi, plants, and mammals (Fig. S5). Notably, *Cs*-Aldhs were distributed across distinct lineages, clustering with either classical Aldh classes (I–III) or microbial aromatic Aldhs. These findings suggest that *Cs*-Aldhs originated from multiple ancestral lineages and may have evolved specialized functions in response to environmental aromatic compounds.

### Three-dimensional Structural Modeling of Cs-Aldhs from C. subvermispora

**Fig. 3:**
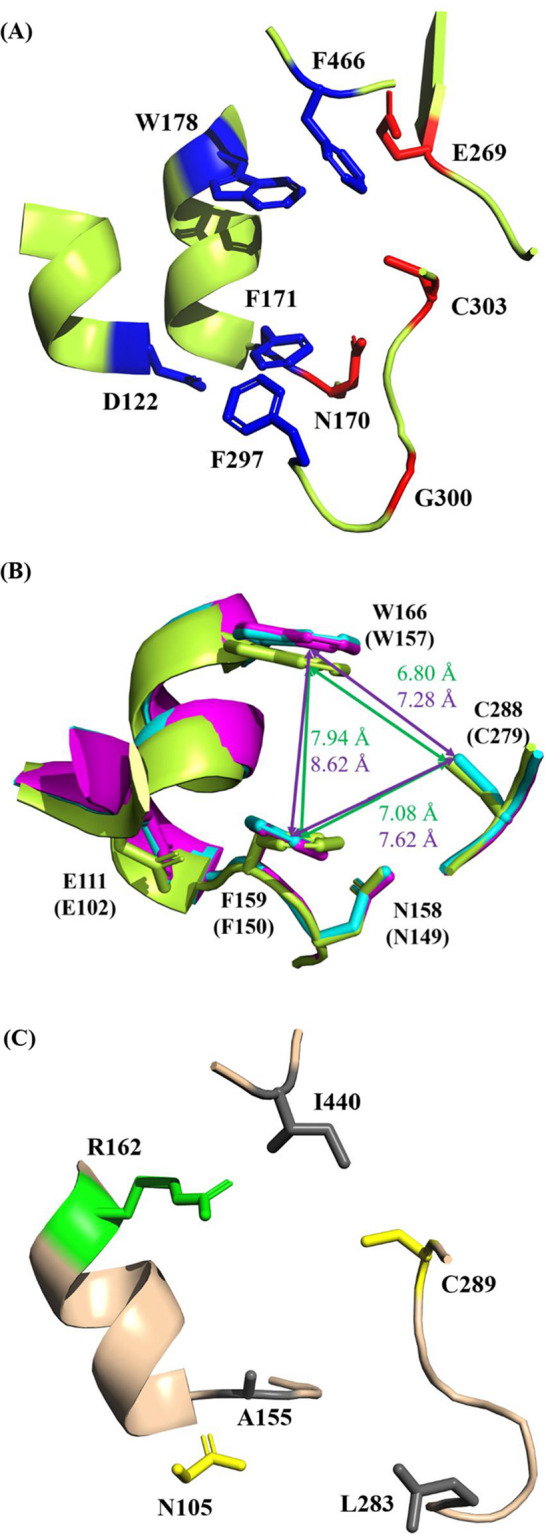
Predicted 3D substrate channels and structural alignments of *Cs*-AldhA, *Cs*-AldhF, *Cs*-AldhH, and *Cs*-AldhJ. Three-dimensional structures were predicted using ColabFold (27–29), which generated five plausible models per *Cs*-Aldh based on full-length amino acid sequences. The resulting structures were visualized in PyMOL (Ver. 2.5) and aligned based on the conserved catalytic cysteine (C303 in *Cs*-AldhA) to compare overall shapes and substrate channel architectures. **(A)** The backbone structure of *Cs*-AldhA is shown in light green. Five residues predicted to interact with the substrate (D122, F171, W178, F297, and F466) are highlighted in blue, while four residues corresponding to the catalytic site (N170, E269, G300, and C303) are colored red (Fig. S3). The side chains of these nine residues are displayed with their sequence positions labeled. **(B)** Structural models of *Cs*-AldhF and *Cs*-AldhH were aligned to *Cs*-AldhA based on the position of C303. For clarity, several residues listed in (A) were omitted in the magnified view. The backbones and side chains are colored light green (*Cs*-AldhA), cyan (*Cs*-AldhF), and magenta (*Cs*-AldhH). Distances between tryptophan, phenylalanine, and cysteine residues are indicated in green (*Cs*-AldhA) and purple (*Cs*-AldhH); corresponding values are listed in Table S4. Reference points for distance measurements are defined as follows: the center of the carbonyl carbon for aspartic acid and glutamic acid; the centroid of the benzene ring for phenylalanine and tryptophan; and the sulfur atom center for cysteine. **(C)** The backbone model of *Cs*-AldhJ is shown in ivory. Side chains of five residues corresponding to the substrate-interacting residues (blue) in (A) are displayed. Nonpolar hydrophobic residues are colored grey, while polar and basic residues are shown in yellow and green, respectively. Single-letter amino acid codes and residue numbers correspond to *Cs*-AldhA (A), *Cs*-AldhF (B), and *Cs*-AldhJ (C); values for *Cs*-AldhH are shown in parentheses in panel (B)

The 3D structures of 16 *Cs*-Aldhs were predicted using ColabFold [27–29] and visualized in PyMOL, with a focus on the residues located within the catalytic center and substrate-binding channel. Sequence analysis (Fig. S3) revealed that *Cs-*AldhA–D possess an aldehyde dehydrogenase 2 (ALDH2)-like domain that includes four aromatic residues and an aspartic acid (D122 in *Cs*-AldhA) surrounding the substrate-binding channel (Fig. 3A; Fig. S3; [35]).

In *Cs*-AldhF and *Cs*-AldhH, this aspartic acid is replaced by glutamic acid (E111 and E102, respectively) (Fig. 3B, Fig. S3). Nevertheless, the distance from these residues to the catalytic cysteine (C288 in *Cs*-AldhF; C279 in *Cs*-AldhH) remains similar to that in *Cs*-AldhA (Table S4, distance *z*). However, two aromatic residues (F159 and W166 in *Cs*-AldhF) are positioned farther apart in *Cs*-AldhF (8.56 Å) and *Cs*-AldhH (8.62 Å) than in *Cs*-AldhA (7.94 Å), and their relative orientations are less parallel. These differences may impair substrate stabilization (Fig. 3B; Table S4, distance *w*). *Cs-*AldhK and *Cs*-AldhP also contain tryptophan and phenylalanine residues but have narrower substrate-binding pockets than *Cs*-AldhA–D (Table S4, distances *w* and *z*).

*Cs*-AldhJ and *Cs*-AldhL–O contain an asparagine (e.g., N105 in *Cs*-AldhJ) instead of an acidic residue at the analogous position (Fig. 3C, Fig. S6). Several key residues—highlighted with white text on a black background in Fig. S3—that contribute to the substrate-binding channel in *Cs*-AldhA–D are not conserved in these enzymes. For example, two bulky aromatic residues (F171 and F297) in *Cs*-AldhA are replaced by smaller hydrophobic residues (A155 and L283) in *Cs*-AldhJ (Figs. 3A and 3C), increasing the distance to the catalytic cysteine (C289 in *Cs*-AldhJ) from 7.08 Å to 8.12 Å (Table S4, distance *x*). Additionally, W178 is substituted with a positively charged arginine (R162) in *Cs*-AldhJ and *Cs*-AldhL–O (Fig. 3C, Fig. S3). Similar substitutions were also observed in *Cs-*AldhE (R173), *Cs*-AldhG (K156), and *Cs*-AldhI (N146), indicating notable variation in this critical region (Fig. S3).

### Heterologous Expression of Cs-Aldhs Using the Rhodococcus Expression System

**Fig. 4:**
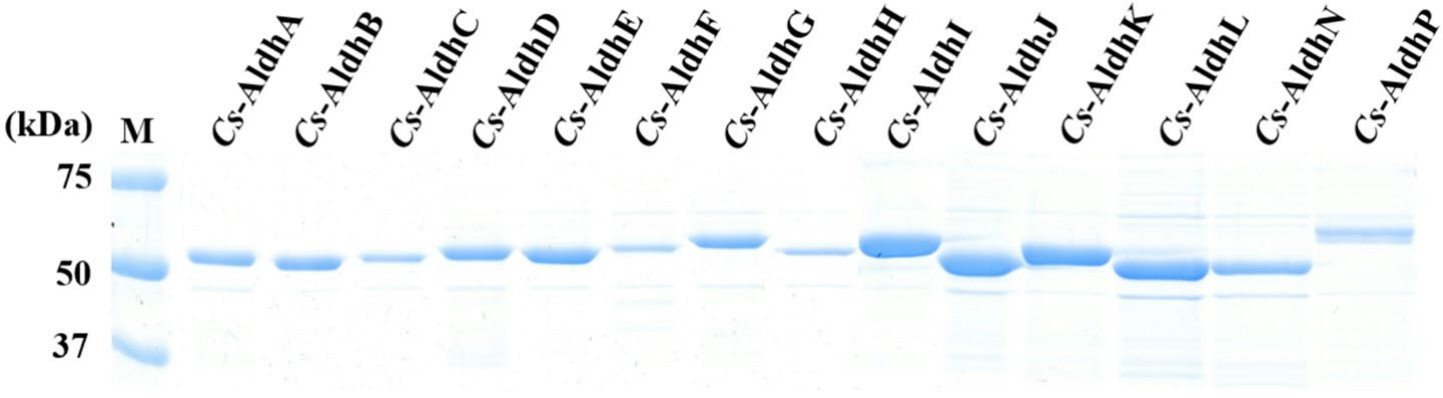
SDS-PAGE analysis of purified His-tagged *Cs*-Aldhs expressed in *R. erythropolis*. Fourteen *Cs*-Aldhs were successfully expressed as soluble His-tagged fusion proteins in *Rhodococcus erythropolis* and purified by affinity chromatography. Distinct bands corresponding to the predicted molecular weights (Table 1) were observed for each purified protein. *Cs*-AldhF was expressed with an N-terminal His-tag, while *Cs*-AldhM and *Cs*-AldhO were excluded due to insolubility. Lane M represents the molecular weight marker

To investigate the functions of the 16 *Cs*-Aldhs, initial attempts were made to express the cloned genes in *Escherichia coli* using the pET vector system [16]. However, soluble expression was not achieved, likely due to extremely low expression levels despite efforts to optimize culture conditions. As an alternative, the genes were expressed in *Rhodococcus erythropolis* L88 using the constructed vectors pTipQT1 or pTipQT2 (Fig. S2). This approach successfully yielded 14 soluble His-tagged fusion proteins (mostly C-terminal tags; N-terminal for *Cs*-AldhF), which were purified via affinity chromatography (Fig. 4). *Cs*-AldhM and *Cs*-AldhO remained insoluble despite attempts with both N- and C-terminal His-tags and low-temperature induction. SDS-PAGE analysis confirmed that the molecular weights of purified proteins matched with the theoretical values based on their amino acid sequences (Table 1), and yields ranged from 0.2 to 4.7 mg per 100 mL culture. These results demonstrate that the *Rhodococcus* expression system is suitable for producing *Cs*-Aldhs from *C. subvermispora*, offering improved solubility and protein yield compared to *E. coli*.

### Evaluation of Cs-Aldh Enzyme Activity Using Fluorescence-based NADH Detection

To evaluate the enzymatic activity of the 14 purified *Cs*-Aldhs (Fig. 4), a fluorescence-based method for detecting NADH was employed. Traditional absorbance-based detection at 340 nm [36] was unsuitable due to spectral overlap with aromatic aldehydes such as vanillin, syringaldehyde (368 nm), and *p*-hydroxybenzaldehyde (330 nm). Similarly, the alternative 400-nm method [16] was ineffective because of the inherently low absorbance of NADH at this wavelength. Instead, a fluorescence-based assay exploiting the strong emission of NADH at 450–460 nm was adopted, providing a 30–100-fold higher signal relative to the substrates and their oxidation products (Fig. S1).

Substrate preference and catalytic efficiency were assessed using four lignin-derived aromatic aldehydes: vanillin, syringaldehyde, protocatechualdehyde, and *p*-hydroxybenzaldehyde. Six representative *Cs*-Aldhs (*Cs*-AldhA, E, H, I, J and K) were selected based on the phylogenetic analysis (Fig. 2), and their specific activities were measured (Table 2). *Cs*-AldhJ exhibited the highest activity across all tested substrates, particularly for vanillin (4.79 ± 0.27 U/mg) and syringaldehyde (5.22 ± 0.05 U/mg). *Cs*-AldhE and *Cs*-AldhH showed high activities toward protocatechualdehyde (4.24 ± 0.78 U/mg) and *p*-hydroxybenzaldehyde (7.67 ± 0.06 U/mg), respectively. In contrast, *Cs*-AldhA and *Cs*-AldhK showed relatively low specific activities.

**Table 2:** Fluorescence intensity rates and specific activities of six representative *Cs*-Aldhs against four lignin-derived aromatic compounds

ΔRFU/Δt				
Enzyme**	Vanillin	Syringaldehyde	Protocatechualdehyde	*p*-Hydroxybenzaldehyde
*Cs*-AldhA	15.1 ± 1.0	4.1 ± 0.4	6.9 ± 0.5	
*Cs*-AldhE	73.1 ± 7.0	3.6 ± 0.5	445.0 ± 44.9	
*Cs*-AldhH	285.1 ± 5.1	156.3 ± 4.2	117.1 ± 1.7	
*Cs*-AldhI	12.6 ± 0.8	94.9 ± 1.5	7.5 ± 0.2	
*Cs*-AldhJ	592.4 ± 25.8	583.4 ± 2.1	260.3 ± 37.8	
*Cs*-AldhK	23.1 ± 0.5	70.3 ± 2.0	43.2 ± 1.3	
Specific activity***				
Enzyme	Vanillin	Syringaldehyde	Protocatechualdehyde	*p*-Hydroxybenzaldehyde
*Cs*-AldhA	0.09 ± 0.01	0.02 ± 0.00****	0.04 ± 0.00	0.03 ± 0.00
*Cs*-AldhE	0.44 ± 0.05	0.02 ± 0.00	4.24 ± 0.78	1.11 ± 0.16
*Cs*-AldhH	1.71 ± 0.03	0.91 ± 0.02	0.66 ± 0.01	7.67 ± 0.06
*Cs*-AldhI	0.08 ± 0.00	0.64 ± 0.00	0.04 ± 0.00	0.03 ± 0.00
*Cs*-AldhJ	4.79 ± 0.27	5.22 ± 0.05	1.86 ± 0.34	4.25 ± 0.81
*Cs*-AldhK	0.13 ± 0.00	0.38 ± 0.01	0.24 ± 0.01	0.05 ± 0.01

*The unit for fluorescence intensity rate (∆RFU/∆t) is min⁻¹.

**Six representative *Cs*-Aldhs, one from each of the six phylogenetic groups identified in the tree (Fig. 2), are listed.

***Specific activity is expressed in units (U·mg⁻¹), where one unit (U) is defined as the amount of enzyme that produces 1 nmol of NADH per minute (nmol·min⁻¹·mg⁻¹).

****Standard deviations shown as “0.00” indicate values below 0.005, rounded to two decimal places.

To further characterize this reduced catalytic efficiency, kinetic analyses of *Cs*-AldhA and *Cs*-AldhK were performed using varying concentrations of vanillin (Fig. S7). *Cs*-AldhA exhibited substrate inhibition, with a maximum rate observed at 0.209 mM. Its kinetics fitted the Haldane model, yielding a *Km* of 0.137 mM, a *Vmax* of 0.000295 mM·min⁻¹, and an inhibition constant (*Ki*) of 0.320 mM. In contrast, *Cs-*AldhK followed classical Michaelis–Menten kinetics, with a *Km* of 0.0578 mM and a *Vmax* of 0.000137 mM·min⁻¹. These parameters are consistent with their relatively low specific activities toward vanillin (0.09 ± 0.01 and 0.13 ± 0.00 U/mg, respectively; Table 2), confirming limited catalytic performance under these conditions.

### Quantitative Analysis of Aromatic Aldehyde Consumption and Aromatic Acid Production by Cs-Aldhs

To more accurately evaluate the catalytic performance of *Cs*-Aldhs, both substrate consumption and product formation were quantified using gas chromatography–mass spectrometry (GC–MS) under the same conditions as the specific activity assays (0.5 mM vanillin or syringaldehyde, 25 °C, 2 h).

The oxidation products—vanillic acid and syringic acid—were detected as *O-*trimethylsilyl (*O-*TMS) derivatives. Fragment ions at *m/z* 223 and 253 likely correspond to the loss of a complete *O*-TMS group from vanillic acid-2TMS and syringic acid-2TMS, respectively (Figs. S8G and S8H). In contrast, the fragment at *m/z* 193 in vanillic acid-2TMS (and *m/z* 223 in syringic acid-2TMS) likely results from a combined cleavage of the *O*-TMS and methoxyl groups from the aromatic ring. These fragmentation patterns were consistent with those of authentic standards (Figs. S8A–F).

**Fig. 5:**
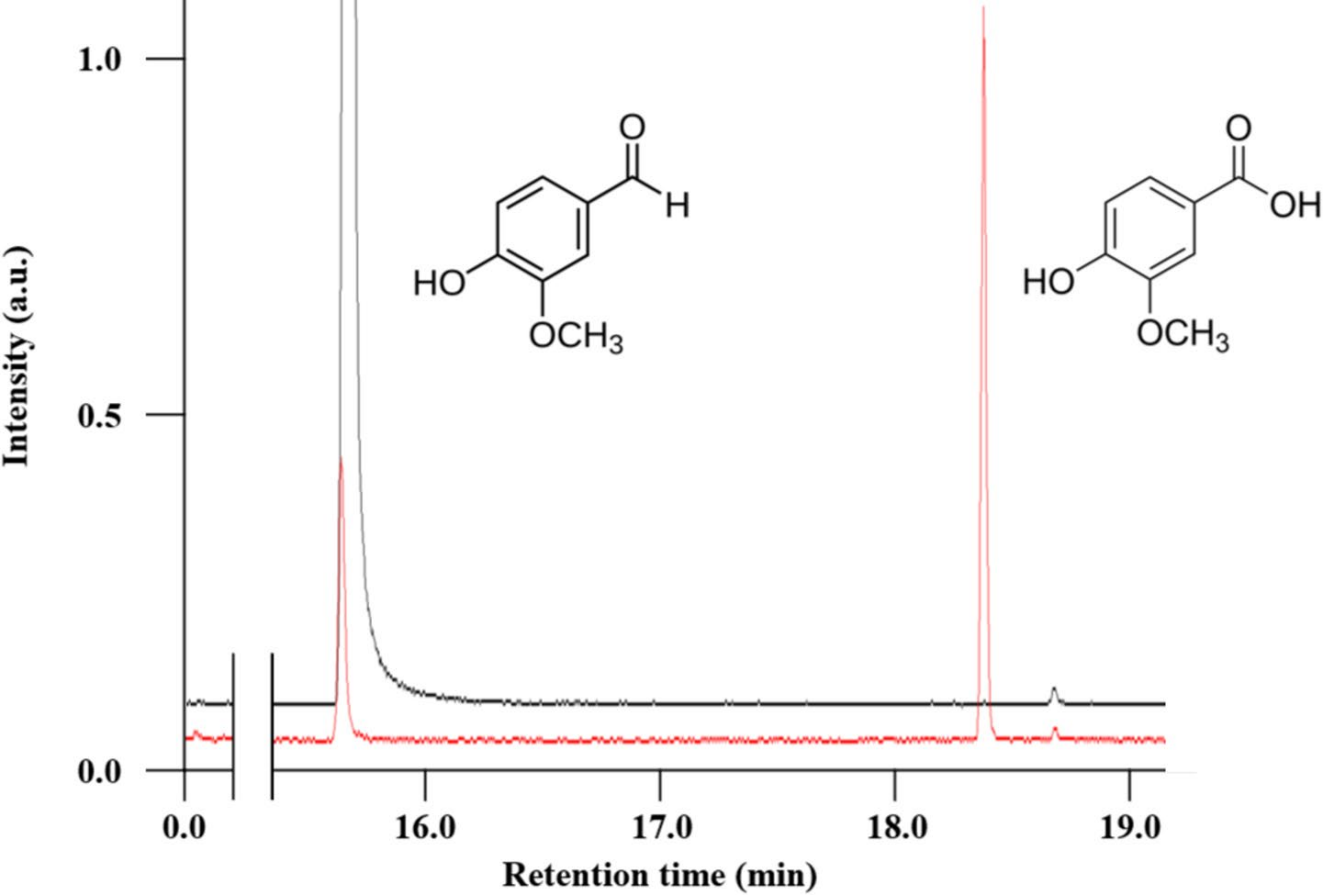
Conversion of vanillin to vanillic acid by purified *Cs*-AldhA. Representative GC–MS chromatograms showing the conversion of vanillin (retention time: 15.57 min) to vanillic acid (18.17 min) by *Cs*-AldhA. Reactions were conducted in 1 mL of 50 mM potassium phosphate buffer [pH 8.0] containing 0.1 mM vanillin. The control reaction (black line) lacked both NAD⁺ and *Cs*-AldhA, while the enzymatic reaction (red line) contained 0.4 mM NAD⁺ and 100 μL of *Cs*-AldhA. Incubation was performed at 28 °C for 12 hours

Importantly, these products were observed in reactions catalyzed by all six tested *Cs*-Aldhs, confirming that each enzyme exhibits aromatic aldehyde-oxidizing activity under the given conditions. To facilitate visualization of product formation by low-activity enzymes, a representative chromatogram of vanillic acid production by *Cs*-AldhA under optimized conditions is shown in Fig. 5, as a complement to the standard assay results in Fig. S8. Notably, distinct vanillic acid peaks were detected in reactions catalyzed by *Cs*-AldhH (Fig. S8C) and *Cs*-AldhJ (Fig. S8E), indicating high catalytic efficiency.

## Discussion

In this study, we identified 16 aromatic aldehyde dehydrogenases (*Cs-*Aldhs) from the genome of *C. subvermispora* using *Pc-*ALDH1 and *Pc-*ALDH2 from *P. chrysosporium* as query sequences. These enzymes exhibited relatively high amino acid identity (65.7–89.7%) to the reference sequences (Table 1) and retained conserved catalytic, cofactor-binding, and oligomerization motifs (Fig. S3). Phylogenetic and structural analyses grouped them into six clades, revealing variations in three-dimensional conformation and substrate-binding pocket architecture. These findings suggest functional diversification in response to lignin-derived aromatic aldehydes (Figs. 2 and 3; Table 2; Fig. S3; Fig. S6; Table S4).

In humans, ALDH2 is responsible for detoxifying lipid peroxidation-derived aldehydes such as 4-hydroxy-2-nonenal (4-HNE) and malondialdehyde (MDA), thereby protecting cells from oxidative stress [37, 38]. ALDH2 also exhibits esterase and reductase activities, broadening its substrate range [14]. Analogously, the *Cs*-Aldhs may contribute not only to aromatic aldehyde degradation but also to the mitigation of oxidative stress. During the early stages of wood decay, *C. subvermispora* produces large amounts of unsaturated fatty acids, such as linoleic acid, which undergo manganese peroxidase (MnP)-mediated peroxidation, generating lipid radicals [39–42]. Aldh-mediated detoxification of these reactive aldehydes may be essential for maintaining cellular viability during ligninolytic activity.

In addition to lipid-derived aldehydes, *C. subvermispora* also encounters lignin-derived aromatic aldehydes such as vanillin. Our genomic analyses identified all 16 *Cs-aldh* genes in strain ATCC 90467, supporting their potential involvement in aromatic aldehyde metabolism. Although the precise regulatory mechanism remains unclear, these results are consistent with the observed enhancement of fungal growth and increased glucose consumption (Figs. 1A and 1B). This unusual response suggests that *C. subvermispora* possesses a unique tolerance and metabolic capacity toward vanillin, distinguishing it from other white-rot fungi that are typically inhibited at similar concentrations.

Phylogenetic analysis (Fig. 2 and S5) clustered the 16 *Cs*-Aldhs into distinct clades. Structural modeling using AlphaFold2 revealed conserved catalytic cores but substantial divergence in substrate-binding regions, suggesting functional specialization (Figs. 3 and S6). While these *in silico* models provide important hypotheses, their accuracy requires experimental validation.

To examine functional variation among *Cs*-Aldhs, representative enzymes from distinct clades were analyzed in detail. *Cs*-AldhA displayed low catalytic activity and substrate inhibition toward vanillin, fitting the Haldane model. This suggests that the standard concentration (0.5 mM) exceeds its optimal substrate levels (Figs. S7A and S8A). Structural modeling predicted an ALDH2-like domain containing aromatic residues (F171, W178, F297) and an acidic residue (D122), positioned to stabilize aromatic aldehydes through π–π stacking, hydrophobic interactions, and hydrogen bonding [35, 43–45]. Such interactions may also account for the unusual kinetic behavior of *Cs*-AldhA, underscoring the need for further validation by X-ray crystallography or other structural approaches.

Whereas *Cs*-AldhA showed low activity and substrate inhibition, *Cs*-AldhF and *Cs*-AldhH exhibited high activity toward *p*-hydroxybenzaldehyde. These enzymes contain a glutamate residue (E111 or E102) instead of D122 (Fig. 3B), and structural modeling revealed increased distances between aromatic residues (Fig. S6, Table S4), potentially influencing substrate recognition. Similarly, *Cs-*AldhE and *Cs*-AldhG, although phylogenetically close to *Cs*-AldhA–D, carried basic residues (R173 or K156) in place of W178 (Fig. S6). This shift from π-stacking to electrostatic interactions may enhance binding to smaller substrates such as protocatechualdehyde. A comparable mechanism has been reported for *Pseudomonas putida* phenylacetaldehyde dehydrogenase, in which substitution of aromatic residues lining the catalytic channel with smaller amino acids narrowed the pocket and restricted the substrate scope to smaller aldehydes [46]. Together, these observations highlight how subtle residue changes in the catalytic channel can critically reshape the balance among substrate size, stabilization, and catalytic efficiency.

**Fig. 6:**
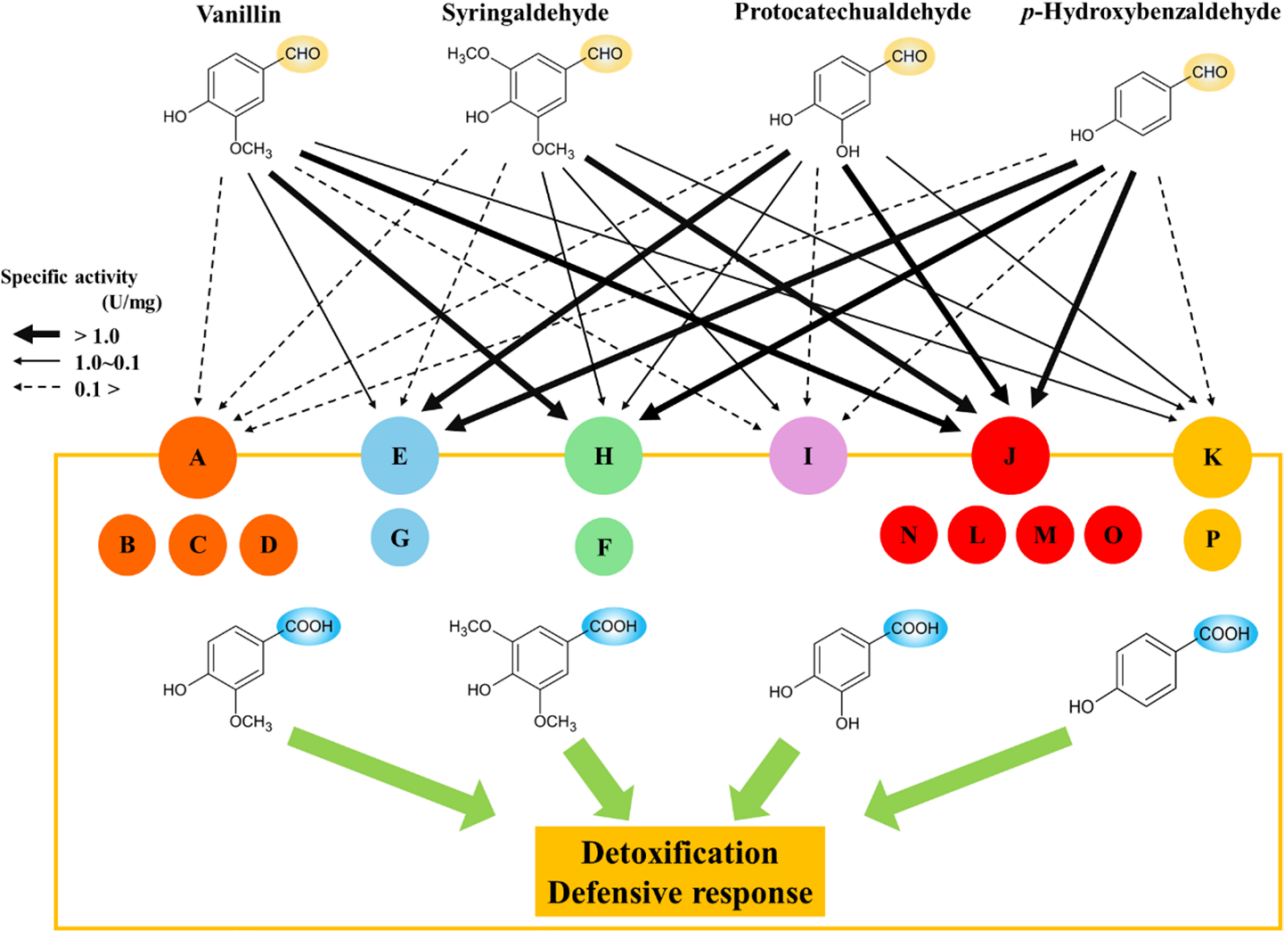
Proposed detoxification and defense response mechanisms against exogenous aromatic aldehydes in *C. subvermispora.* Each capital letter (A–P) corresponds to one of the 16 *Cs-*Aldhs (*Cs*-AldhA–*Cs*-AldhP), grouped according to the phylogenetic classification shown in Fig. 2. The six enzymes analyzed in detail in this study (*Cs*-AldhA, E, H, I, J, and K) are indicated by larger circles. Circle colors represent the clades defined in the phylogenetic tree, and arrow thickness denotes the relative specific activity of each enzyme, as reported in Table 2. This schematic model integrates phylogenetic grouping with enzymatic properties to illustrate the proposed roles of *Cs*-Aldhs in aldehyde detoxification and defense

* Cs*-AldhI, an early-branching enzyme, possessed a Y139–N146 pair near its binding pocket (Fig. S6). Although its overall activity was low, it displayed higher specificity for syringaldehyde (Fig. 6), possibly reflecting favorable electrostatic interactions. *Cs*-AldhJ demonstrated broad substrate preference and high specific activity (Fig. 6). The replacement of W178 with R162 and F171 with A155 likely widened the binding pocket and introduced new electrostatic features. A comparable principle has been demonstrated in plant ALDH10s, where residue substitutions modified the electrostatic environment and cavity size of the catalytic channel, resulting in marked changes in substrate range [47]. Its phylogenetic proximity to bacterial vanillin dehydrogenases (Fig. 2 and S5) suggests a potential evolutionary relationship with bacterial counterparts. Indeed, horizontal gene transfer from bacteria to fungi has been documented in other systems [48]; however, additional evidence would be required to support this possibility.

*Cs*-AldhK also exhibited low activity but followed Michaelis–Menten kinetics (Fig. S7B). Unlike *Cs*-AldhA, it lacks an ALDH2-like domain and shows homology to methylmalonate-semialdehyde dehydrogenases (Fig. 2), which typically form tetramers. Structural modeling revealed a narrow substrate-binding pocket (Fig. S6, Table S4). Its high substrate affinity (*Km* = 0.0578 mM) but low *Vmax* suggest that catalytic turnover may be limited by restricted substrate access or by incomplete oligomerization in the heterologous expression system. As shown in Fig. 1, vanillin exposure promoted both glucose consumption and fungal growth, suggesting that aldehyde detoxification is linked to enhanced primary metabolism. Consistent with this view, GC–MS analysis confirmed that vanillin was degraded in *C. subvermispora* cultures but remained largely undegraded in *P. chrysosporium* under identical conditions (Fig. S4), providing direct evidence that the enhanced growth of *C. subvermispora* is associated with active vanillin catabolism. In line with previous reports on vanillin responses in white-rot fungi [15, 16], these findings support the working model presented in Fig. 6, in which distinct substrate affinities and activities among *Cs*-Aldhs contribute to a coordinated enzymatic system that mitigates exogenous aromatic aldehyde toxicity during ligninolysis.

Taken together, our structural, kinetic, and phylogenetic analyses indicate that the *Cs*-Aldh family has undergone functional diversification to accommodate a wide spectrum of lignin-derived aldehydes across varying concentration ranges. *Cs*-AldhA, with aromatic and acidic residues positioned around its catalytic pocket, stabilizes vanillin but undergoes substrate inhibition. *Cs*-AldhK exhibits high substrate affinity yet limited turnover, likely due to its narrow binding pocket. In contrast, *Cs*-AldhJ demonstrates broad substrate specificity and high catalytic efficiency, enabled by residue substitutions that expand the pocket and introduce electrostatic interactions. This functional diversity likely equips *C. subvermispora* with enhanced metabolic flexibility during ligninolytic activity. While AlphaFold2-based modeling provides mechanistic hypotheses, further structural validation—such as X-ray crystallography or cryo-electron microscopy (cryo-EM)—will be essential to confirm these interpretations.

## Supplementary Information

Below is the link to the electronic supplementary material.

Supplementary Material 1 (DOCX 4.91 MB)

## Data Availability

The datasets generated during and/or analyzed during the current study are available from the corresponding author on reasonable request.
