# Correction to: Diversity of Aromatic Aldehyde Dehydrogenases in *Ceriporiopsis subvermispora*: Insights Into Fungal Vanillin Metabolism

**DOI:** 10.1007/s12010-025-05482-z

**Published:** 2025-12-12

**Authors:** Junseok Lee, Takahito Watanabe, Naoko Kobayashi, Ayako Kido, Takashi Watanabe

**Affiliations:** Laboratory of Biomass Conversion, Research Institute for Sustainable Humanosphere, Kyoto University, Gokasho, Uji, Kyoto 611-0011 Japan

**Correction to: Applied Biochemistry and Biotechnology**

 10.1007/s12010-025-05456-1

The original online version of the article was revised. The following errors are as follows:

The original version of this article contained a production error in Table 2.

Specifically, the entire “*p*-Hydroxybenzaldehyde” column in the ΔRFU/Δt section was inadvertently omitted during the production process, despite being correctly included in both the accepted manuscript and the author proof.

Original:


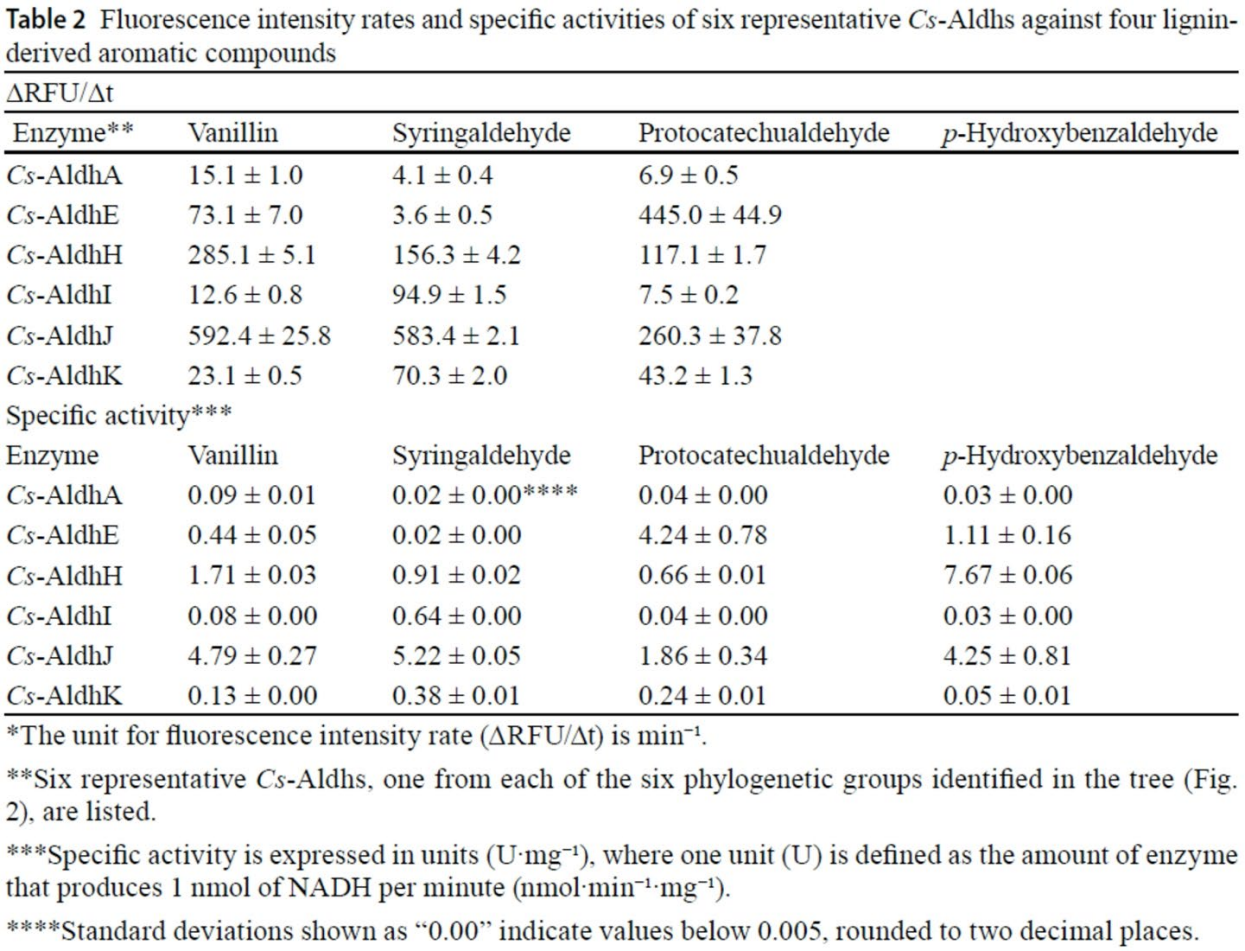


Correct version:

**Table 2:** Fluorescence intensity rates and specific activities of six representative *Cs*-Aldhs against four lignin-derived aromatic compounds

∆RFU/∆t*				
Enzyme**	Vanillin	Syringaldehyde	Protocatechualdehyde	*p*-Hydroxybenzaldehyde
*Cs*-AldhA	15.1 ± 1.0	4.1 ± 0.4	6.9 ± 0.5	5.6 ± 0.3
*Cs*-AldhE	73.1 ± 7.0	3.6 ± 0.5	445.0 ± 44.9	170.7 ± 20.3
*Cs*-AldhH	285.1 ± 5.1	156.3 ± 4.2	117.1 ± 1.7	1121.0 ± 9.3
*Cs*-AldhI	12.6 ± 0.8	94.9 ± 1.5	7.5 ± 0.2	6.4 ± 0.1
*Cs*-AldhJ	592.4 ± 25.8	583.4 ± 2.1	260.3 ± 37.8	404.9 ± 55.2
*Cs*-AldhK	23.1 ± 0.5	70.3 ± 2.0	43.2 ± 1.3	8.9 ± 1.1
Specific activity***				
Enzyme	Vanillin	Syringaldehyde	Protocatechualdehyde	*p*-Hydroxybenzaldehyde
*Cs*-AldhA	0.09 ± 0.01	0.02 ± 0.00****	0.04 ± 0.00	0.03 ± 0.00
*Cs*-AldhE	0.44 ± 0.05	0.02 ± 0.00	4.24 ± 0.78	1.11 ± 0.16
*Cs*-AldhH	1.71 ± 0.03	0.91 ± 0.02	0.66 ± 0.01	7.67 ± 0.06
*Cs*-AldhI	0.08 ± 0.00	0.64 ± 0.00	0.04 ± 0.00	0.03 ± 0.00
*Cs*-AldhJ	4.79 ± 0.27	5.22 ± 0.05	1.86 ± 0.34	4.25 ± 0.81
*Cs*-AldhK	0.13 ± 0.00	0.38 ± 0.01	0.24 ± 0.01	0.05 ± 0.01

*The unit for fluorescence intensity rate (∆RFU/∆t) is min⁻¹

**Six representative *Cs*-Aldhs, one from each of the six phylogenetic groups identified in the tree (Fig. 2), are listed

***Specific activity is expressed in units (U·mg⁻¹), where one unit (U) is defined as the amount of enzyme that produces 1 nmol of NADH per minute (nmol·min⁻¹·mg⁻¹)

****Standard deviations shown as “0.00” indicate values below 0.005, rounded to two decimal places

