# Supplementary material for: Diversity of Aromatic Aldehyde Dehydrogenases in Ceriporiopsis subvermispora: Insights into Fungal Vanillin Metabolism
Source: Appl Biochem Biotechnol. 2025 Nov 11;198(1):472–91. doi: 10.1007/s12010-025-05456-1 (PMC12894122; doi:10.1007/s12010-025-05456-1)
Supplement: Supplementary file 1 — Supplementary Material 1 (DOCX 4.91 MB) [file 12010_2025_5456_MOESM1_ESM.docx]

**Supplementary Information**

**Title: Diversity of aromatic aldehyde dehydrogenases in *Ceriporiopsis subvermispora*: Insights into fungal vanillin metabolism**

Authors: Junseok Lee, Takahito Watanabe*, Naoko Kobayashi, Ayako Kido, and Takashi Watanabe

Affiliation: Laboratory of Biomass Conversion, Research Institute for Sustainable Humanosphere, Kyoto University, Gokasho, Uji, Kyoto 611-0011, Japan

*Corresponding author. E-mail: takahito@rish.kyoto-u.ac.jp

**Document Contents:**

**Fig. S1: Fluorescence calibration curve for NADH quantification.**

**Fig. S2. Physical maps of *Rhodococcus* expression vectors used for heterologous expression of *Cs*-Aldhs.**

**Fig. S3. Multiple sequence alignment of 16 Aldhs from *C. subvermispora* ATCC 90467 (*Cs*-AldhA–*Cs*-AldhP) and two aromatic Aldhs from *P. chrysosporium* (*Pc*-Aldh1 and *Pc*-Aldh2).**

**Fig. S4. Gas chromatograms confirming vanillin degradation in liquid cultures of *C. subvermispora* ATCC 90467 and *P. chrysosporium* ATCC 34541.**

**Fig. S5. Expanded phylogenetic analysis of 16 *Cs*-Aldhs and representative Aldhs from diverse taxa.**

**Fig. S6. Predicted 3D models of *Cs*-Aldh substrate-binding channels generated by ColabFold and visualized in PyMOL.**

**Fig. S7. Kinetic analyses of *Cs*-AldhA and *Cs*-AldhK toward vanillin.**

**Fig. S8. GC-MS analysis of enzymatic reaction mixtures of six *Cs*-Aldhs with vanillin and syringaldehyde.**

**Table S1. Media compositions for fungal culture.**

**Table S2: Primer sequences used in this study.**

**Table S3: Transmembrane regions of *Cs*-Aldhs predicted by the SOSUI and TMHMM algorithms.**

**Table S4: Calculated distances between side chains of each *Cs*-Aldh.**

**(A)**


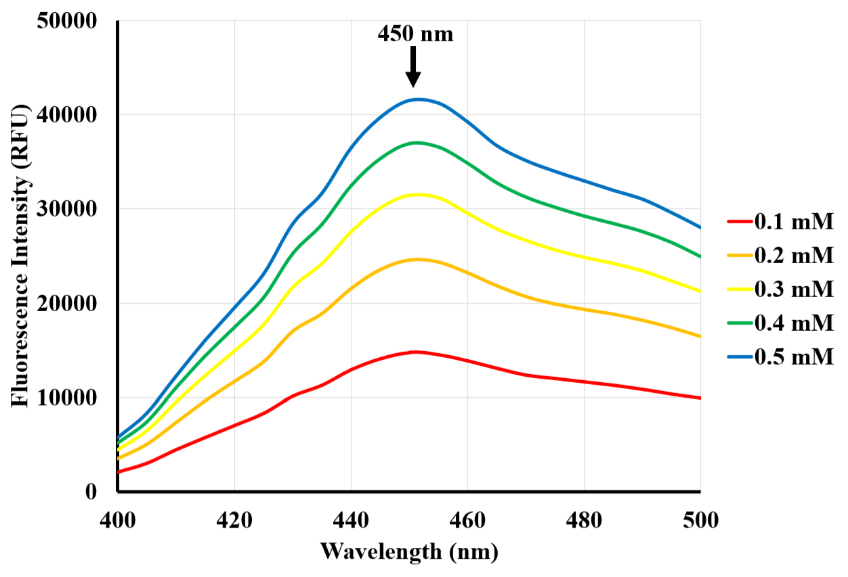


**(B)**


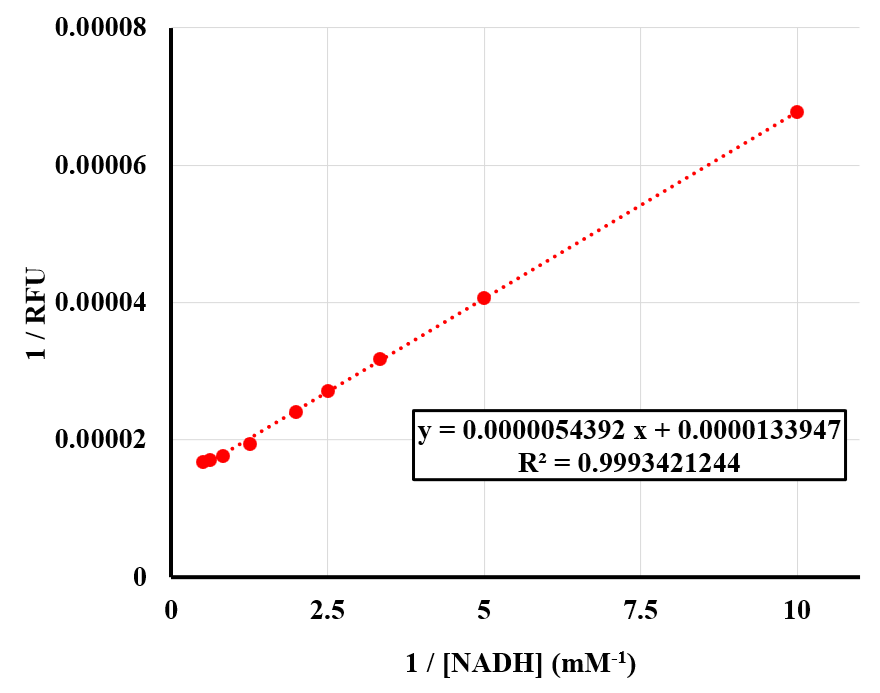


**Fig. S1. Fluorescence calibration curve for NADH quantification.**

**(A)** Emission spectra of NADH at various concentrations (0–0.5 mM) were recorded with excitation at 340 nm. Fluorescence intensity at 450 nm (indicated by black arrow) was used for quantification.

**(B)** A double-reciprocal plot of NADH concentration ([NADH]) versus fluorescence intensity (RFU) produced the following linear regression equation:

1/RFU = 5.44 × 10⁻⁶ / [NADH] + 1.339 × 10⁻⁵

This was rearranged to estimate NADH concentration from RFU values:

[NADH] = (0.4063 × RFU) / (74682.6 − RFU)

This equation was used to calculate NADH production (Δ[NADH]/Δt) and determine enzyme specific activity.


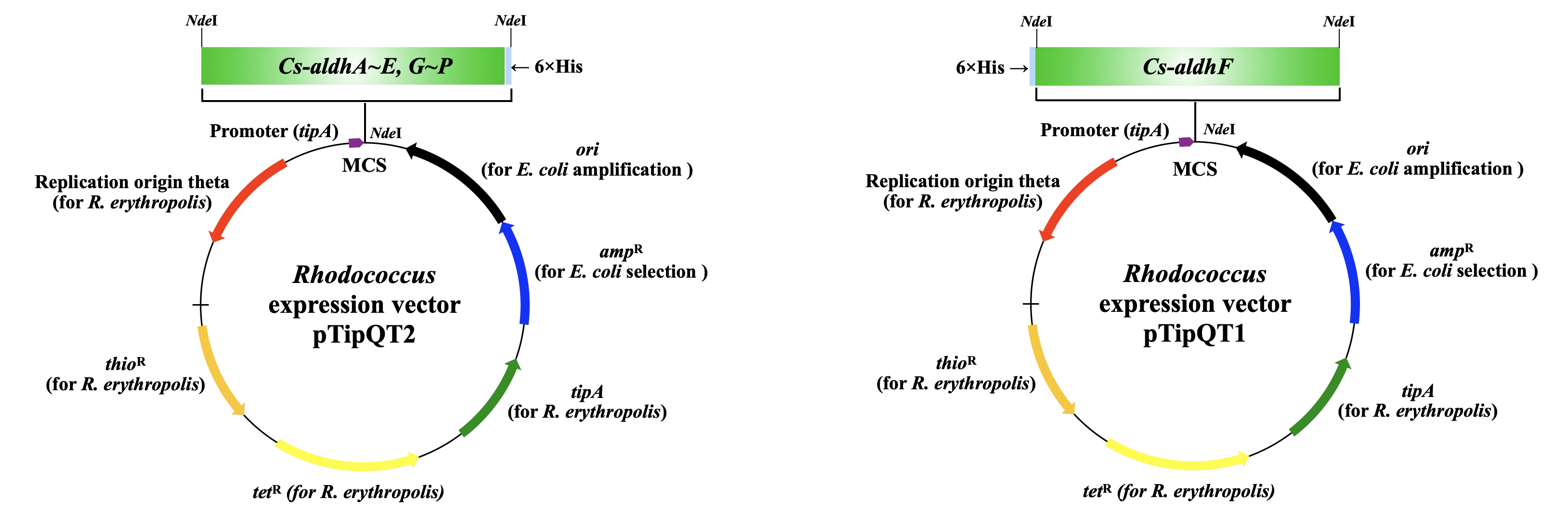


**Fig. S2. Physical maps of *Rhodococcus* expression vectors used for heterologous expression of *Cs*-Aldhs.**

Each *Cs*-Aldh gene was amplified using the primers listed in Table S2 and inserted in the forward orientation into *Nde*I site of the multiple cloning site (MCS) of either pTipQT1 (GenBank accession no. AB127592.1) or pTipQT2 (GenBank accession no. AB127593.1), downstream of the thiostrepton-inducible *tipA* promoter. All constructs featured a C-terminal His-tag (6×His), except for *Cs*-AldhF, which was amplified using the primers *Cs*-*aldhF*.cds_F and *Cs-aldhF*.cdsN_R and cloned to generate an N-terminal His-tag in pTipQT1. Abbreviations used: *thio*^R^, thiostrepton resistance gene; *tet*^R^, tetracycline resistance gene; *tipA*, a gene encoding thiostrepton‑inducible proteins that activate *tipA* promoter and enhance target gene transcription; *amp*^R^, ampicillin resistance gene; *ori*, origin of replication.


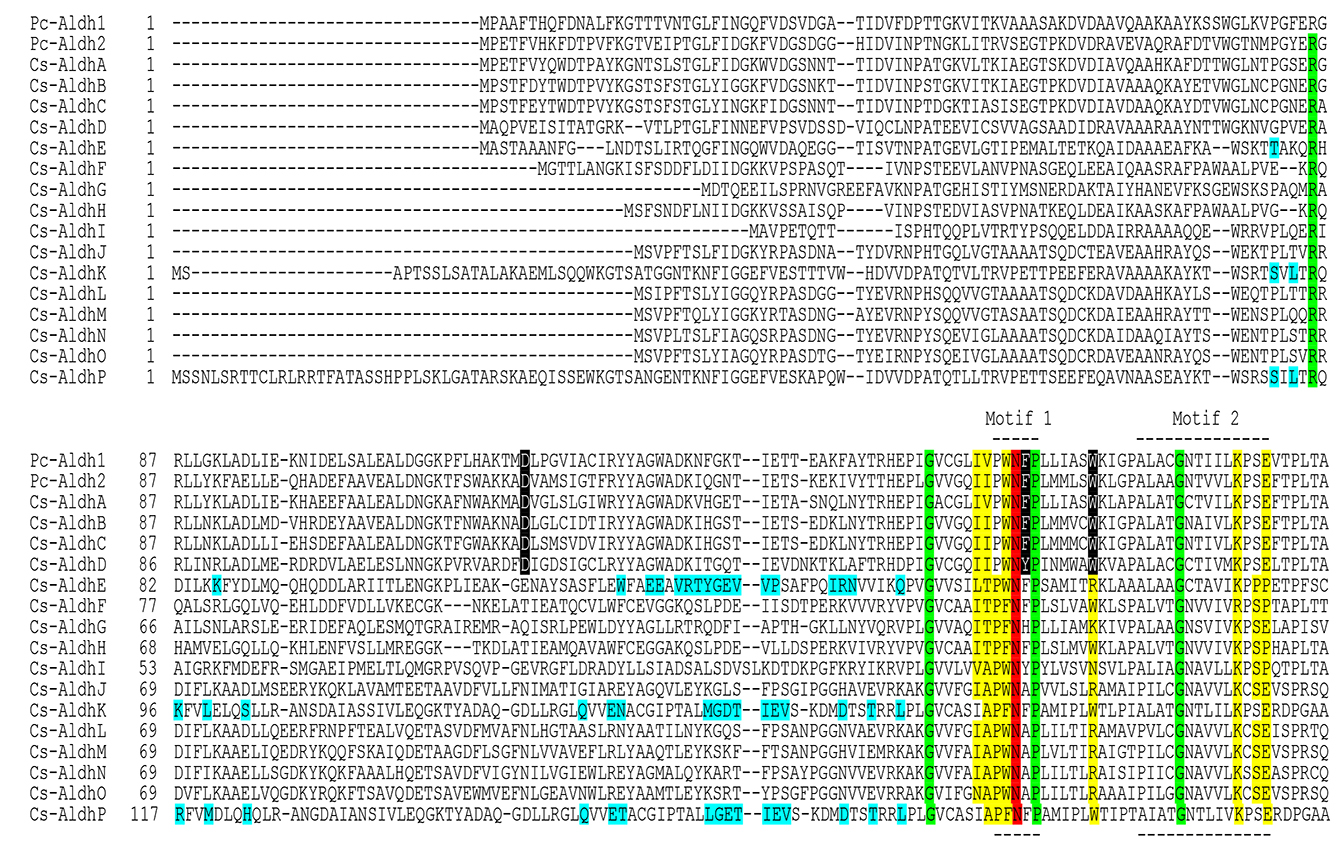


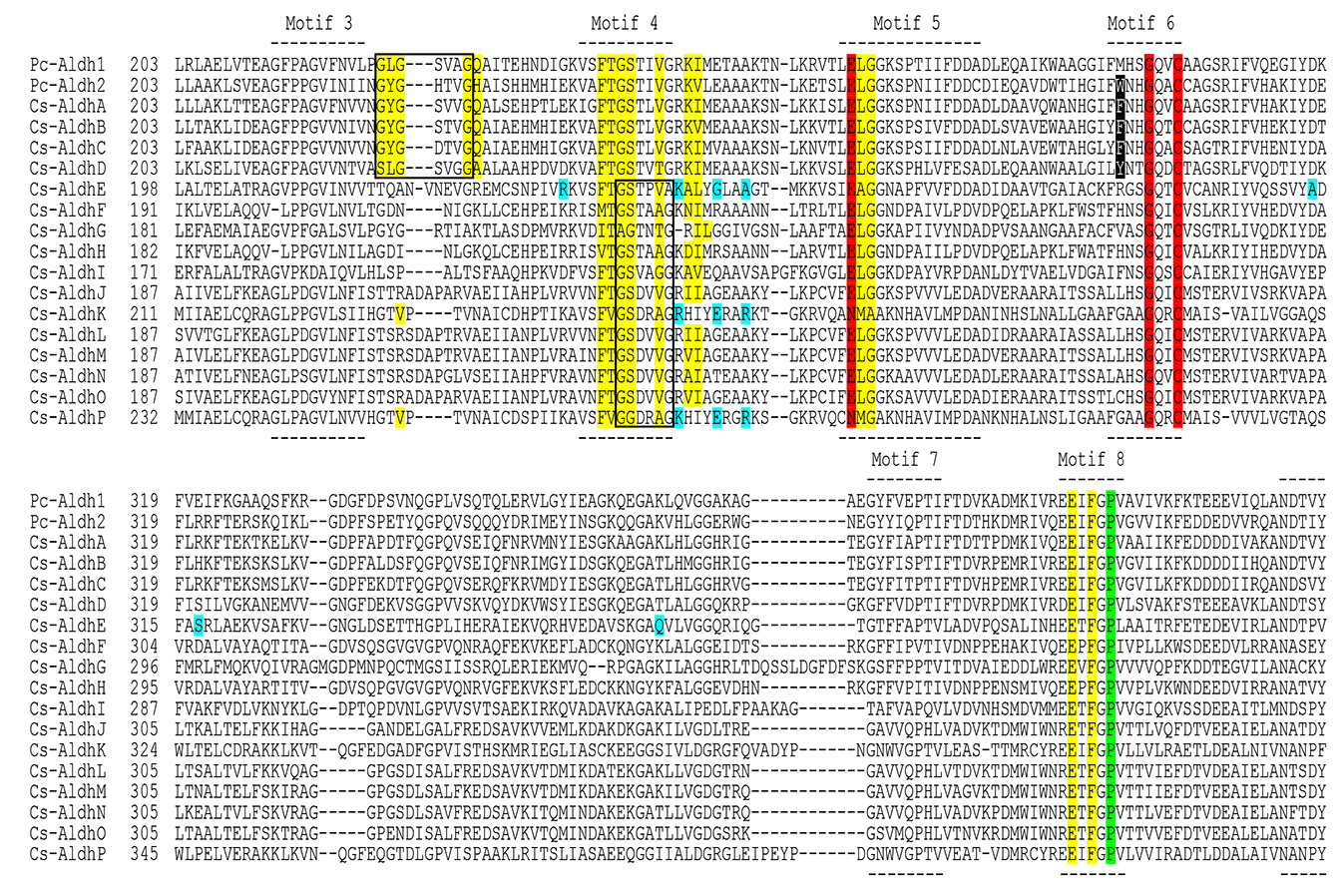


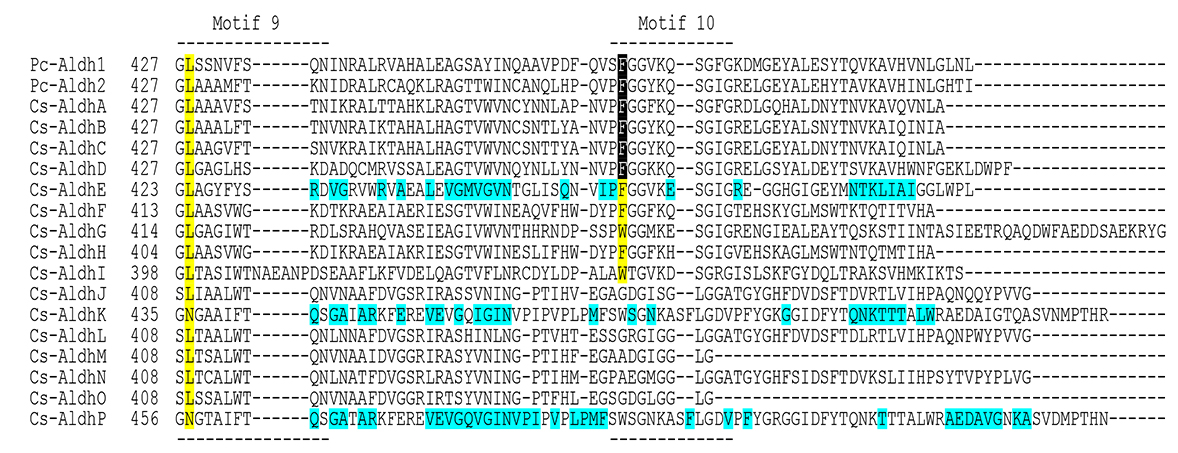


**Fig. S3. Multiple sequence alignment of 16 Aldhs from *C. subvermispora* ATCC 90467 (*Cs*-AldhA–*Cs*-AldhP) and two aromatic Aldhs from *P. chrysosporium* (*Pc*-Aldh1 and *Pc*-Aldh2).** Conserved Aldh motifs (Motifs 1–10) are underlined. Putative active-site residues and coenzyme-binding residues are highlighted in red and yellow, respectively. Residues likely involved in tetramer formation are shown in cyan. Highly conserved residues (>95% identity across all aligned sequences) are shaded in green. Five amino acids predicted to form the substrate-binding channel in *Pc-*Aldh1, *Pc-*Aldh2, and *Cs-*AldhA–D are displayed as white letters on a black background. Boxed regions indicate NAD^+^-binding domains containing the consensus motif GXGXXG or GXXXXG. Notable motif variations are observed in several *Cs*-Aldhs: *Cs*-AldhI exhibits an extended Motif 9; *Cs*-AldhM and *Cs*-AldhO have partially truncated Motif 10; and *Cs*-AldhK and *Cs*-AldhP possess an elongated Motif 10.

1. **(B)**


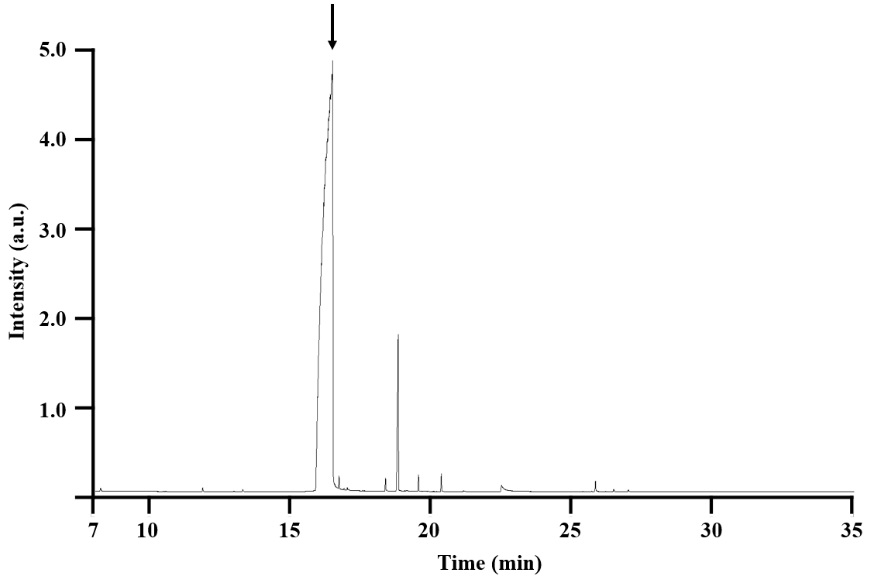

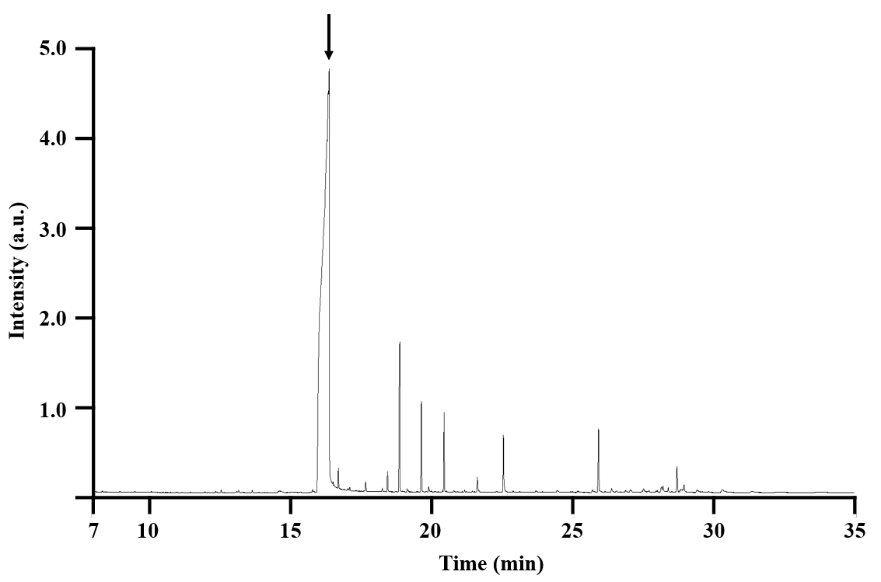


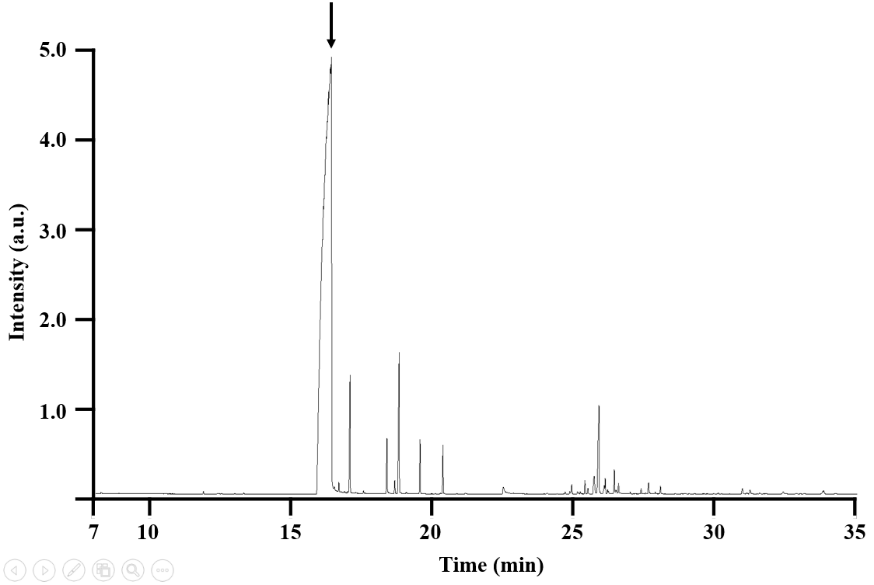

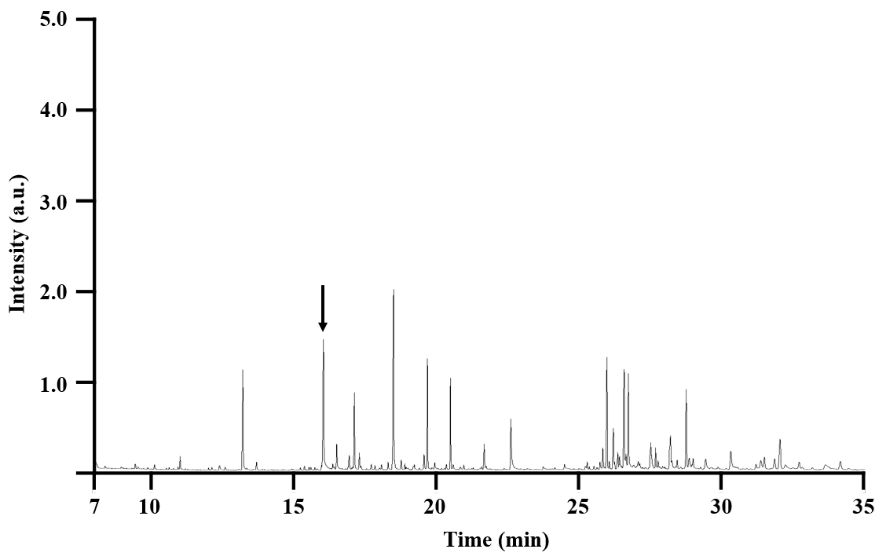


**(C)**


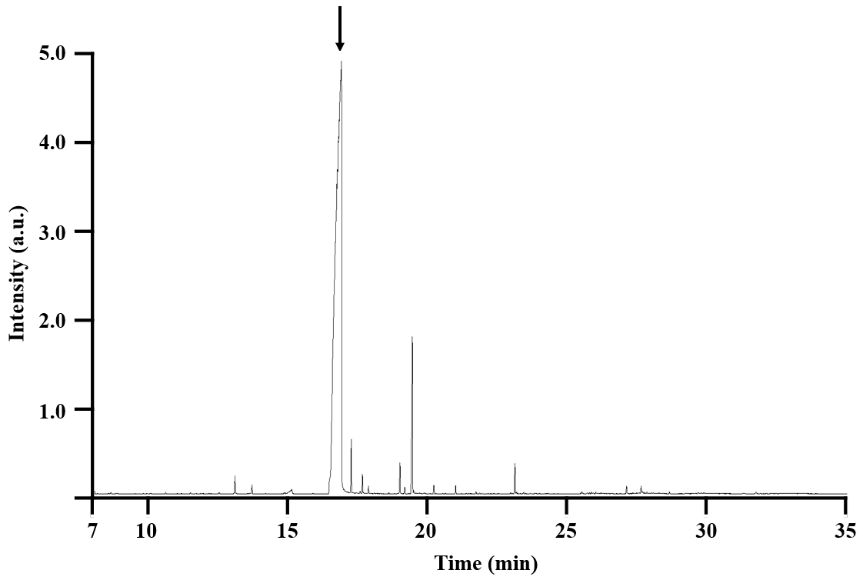


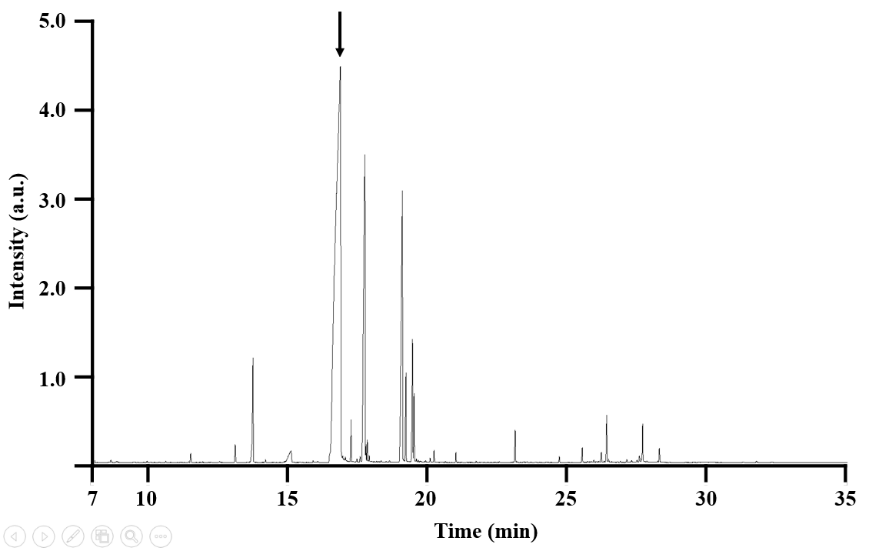


**Fig. S4. Gas chromatograms confirming vanillin degradation in liquid cultures of *C. subvermispora* ATCC 90467 and *P. chrysosporium* ATCC 34541.**

Culture supernatants were collected after removal of mycelia by filtration through Miracloth (25 µm pore size) and analyzed by GC–MS (GCMS-QP2010, Shimadzu, Kyoto, Japan) equipped with a DB-5MS column (30 m × 0.25 mm × 0.25 µm; Agilent) operated in electron ionization (EI) mode. Organic compounds were extracted twice with an equal volume of ethyl acetate and derivatized with MSTFA containing 1% TMCS (Thermo Fisher Scientific, Waltham, MA, USA). The retention time of vanillin (ca. 15.8 min) is indicated by arrows. The upper chromatogram represents the medium immediately after addition of 2 mM vanillin, whereas the lower chromatogram shows the medium collected after 3 days of cultivation. **(A)** Filtered BIII medium supplemented directly with 2 mM vanillin without pre-cultivation. **(B)** Filtered BIII medium after 1 week of pre-cultivation prior to vanillin addition. **(C)** The same experimental setup as in (B), but using *P. chrysosporium*. Chromatographic conditions followed a previously described protocol ***(31)***.


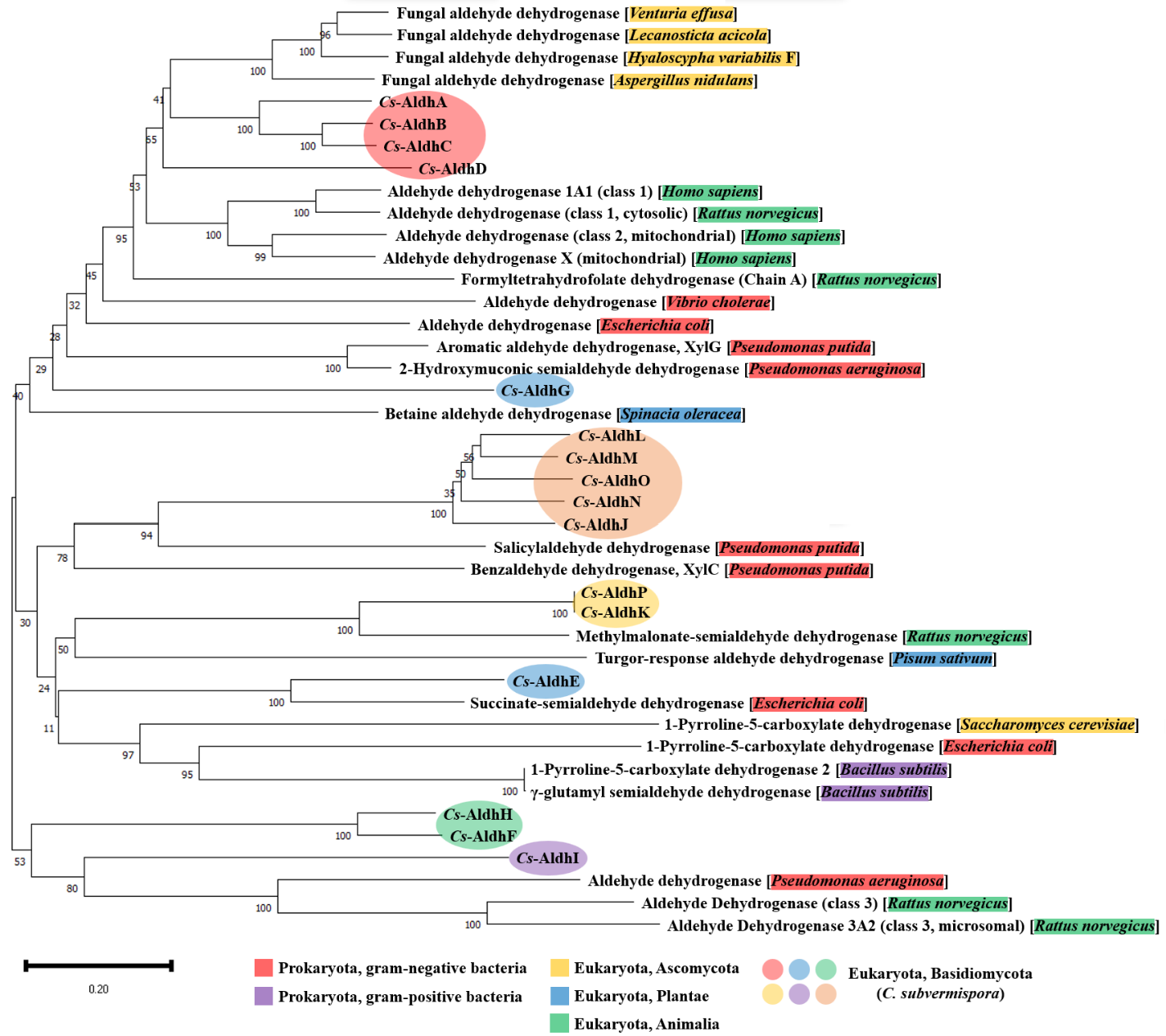


**Fig. S5. Expanded phylogenetic analysis of 16 *Cs*-Aldhs and representative Aldhs from diverse taxa.**

To explore broader evolutionary relationships, a neighbor-joining phylogenetic tree was constructed using ClustalW-aligned amino acid sequences ***(25)*** and validated by 10,000 bootstrap replications using MEGA version 11.0.13 ***(26)***. This tree includes Aldhs from bacteria, Ascomycota fungi, plants, and mammals, representing well-characterized Aldh classes (Class 1–3), as well as enzymes involved in aromatic compound metabolism.

Sixteen *Cs*-Aldhs (*Cs*-AldhA–P) are marked with colored circles, corresponding to the clade colors used in Fig. 2. Notably, *Cs*-AldhE and *Cs*-AldhG (blue circles) were placed in distinct clades in this expanded tree, in contrast to their clustering in the fungal-only phylogeny (Fig. 2).

Taxonomic groupings are indicated by colored boxes. For clarity, the scientific name of *C. subvermispora* (Basidiomycota, Kingdom Fungi, Eukaryota) is shown only in the legend.

**Representative Aldhs included in the analysis:**

**Class 1 Aldhs:**

• *Homo sapiens*, Aldh 1A1 (NP_000680.2)

• *Rattus norvegicus*, Class 1 cytosolic Aldh (NP_058968.15)

**Class 2 Aldhs:**

• *Homo sapiens*, mitochondrial aldehyde dehydrogenase (CAG33272.1)

• *Homo sapiens*, AldhX (mitochondrial) (PDB ID: 7MJC_A)

**Class 3 Aldhs:**

• *Rattus norvegicus*, microsomal Aldh 3A2 (P30839.1)

• *Rattus norvegicus*, Class 3 Aldh (PDB ID: 1AD3_A)

**Ascomycota fungal Aldhs** (as cited in Perozich *et al*. ***(33)***)**:**

• *Venturia effusa* (QDS74652.1)

• *Lecanosticta acicola* (CAK4031451.1)

• *Hyaloscypha variabilis* F (PMD35284.1)

• *Aspergillus nidulans* (AAA33293.1)

**Bacterial Aldhs** (also cited in ***(33)***):

• *Vibrio cholerae* (AAF93982.1)

• *Escherichia coli* (HAU9392353.1)

• *Pseudomonas aeruginosa* (MCS9555839.1)

**Aldhs involved in aromatic compound metabolism:**

• *Pseudomonas aeruginosa*, 2-hydroxymuconic semialdehyde dehydrogenase (HBN9860459.1)

• *Pseudomonas putida*, aromatic Aldh XylG (P23105.1)

• *Pseudomonas putida*, salicylaldehyde dehydrogenase (ACP74208.2)

• *Pseudomonas putida*, benzaldehyde dehydrogenase XylC (P43503.1)

**Other functionally diverse Aldhs:**

• *Rattus norvegicus*, formyltetrahydrofolate dehydrogenase (2O2P_A)

• *Spinacia oleracea*, betaine Aldh (EC 1.2.1.8) (ACM67311.1)

• *Rattus norvegicus*, methylmalonate-semialdehyde dehydrogenase (Q02253.1)

• *Pisum sativum*, NAD⁺-dependent Aldh (EC 1.2.1.3) (NP_001414662.1)

• *Escherichia coli*, succinate-semialdehyde dehydrogenase (STK10060.1)

• *Saccharomyces cerevisiae*, 1-pyrroline-5-carboxylate dehydrogenase (AAA34924.1)

• *Escherichia coli*, 1-pyrroline-5-carboxylate dehydrogenase (AAB59985.1)

• *Bacillus subtilis*, 1-pyrroline-5-carboxylate dehydrogenase (CAF1848751.1)

• *Bacillus subtilis*, γ-glutamyl semialdehyde dehydrogenase (TKJ14556.1)

**Accession number formats:**

• Entries beginning with two letters (*e.g.*, NP_) represent NCBI Reference Sequences

• Entries beginning with three letters (*e.g*., CAG_) are GenBank entries

• Four-character alphanumeric codes with an underscore and chain ID (*e.g*., 7MJC_A) represent PDB IDs

• Entries starting with one letter followed by five digits (*e.g.*, P30839.1) correspond to UniProt entries


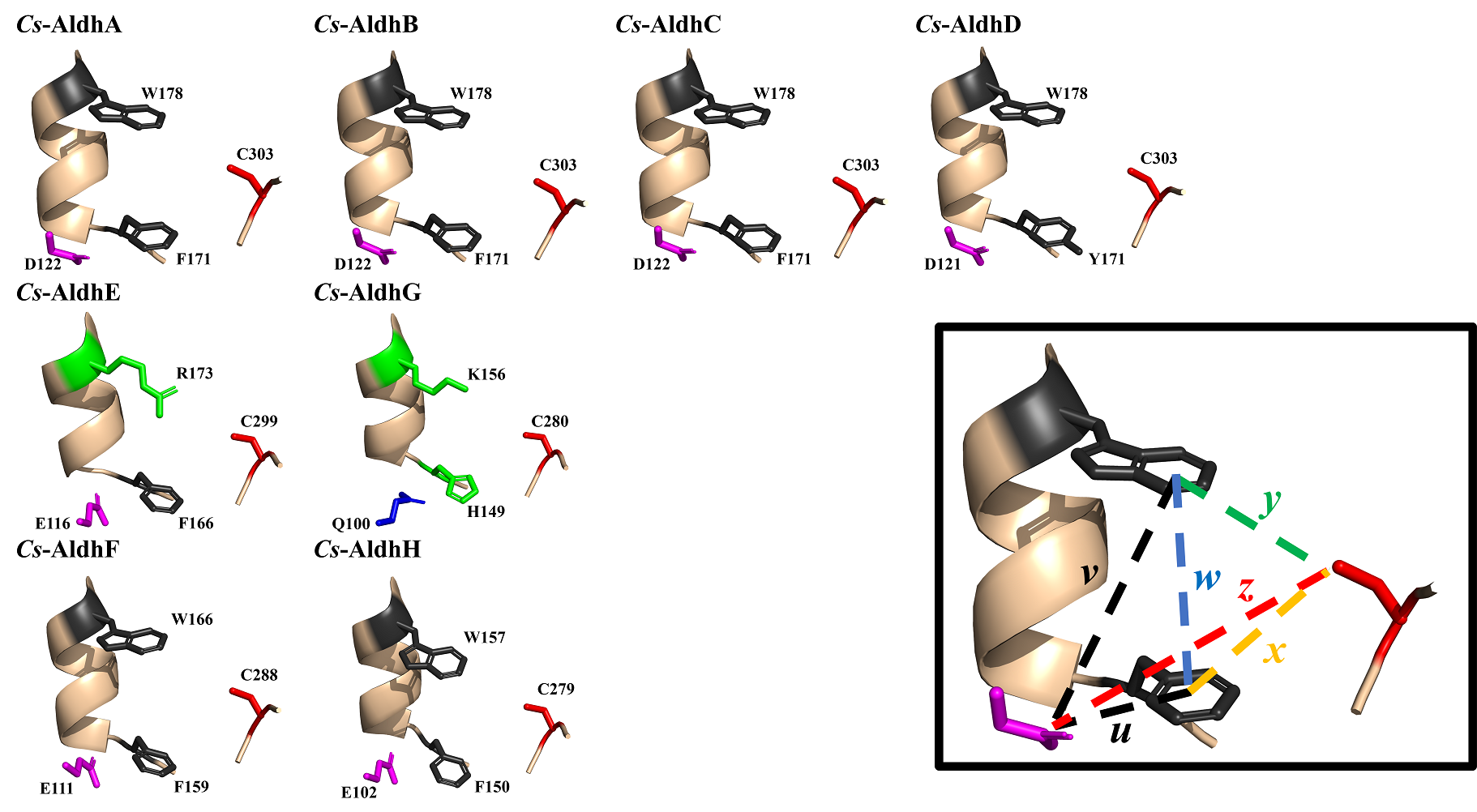


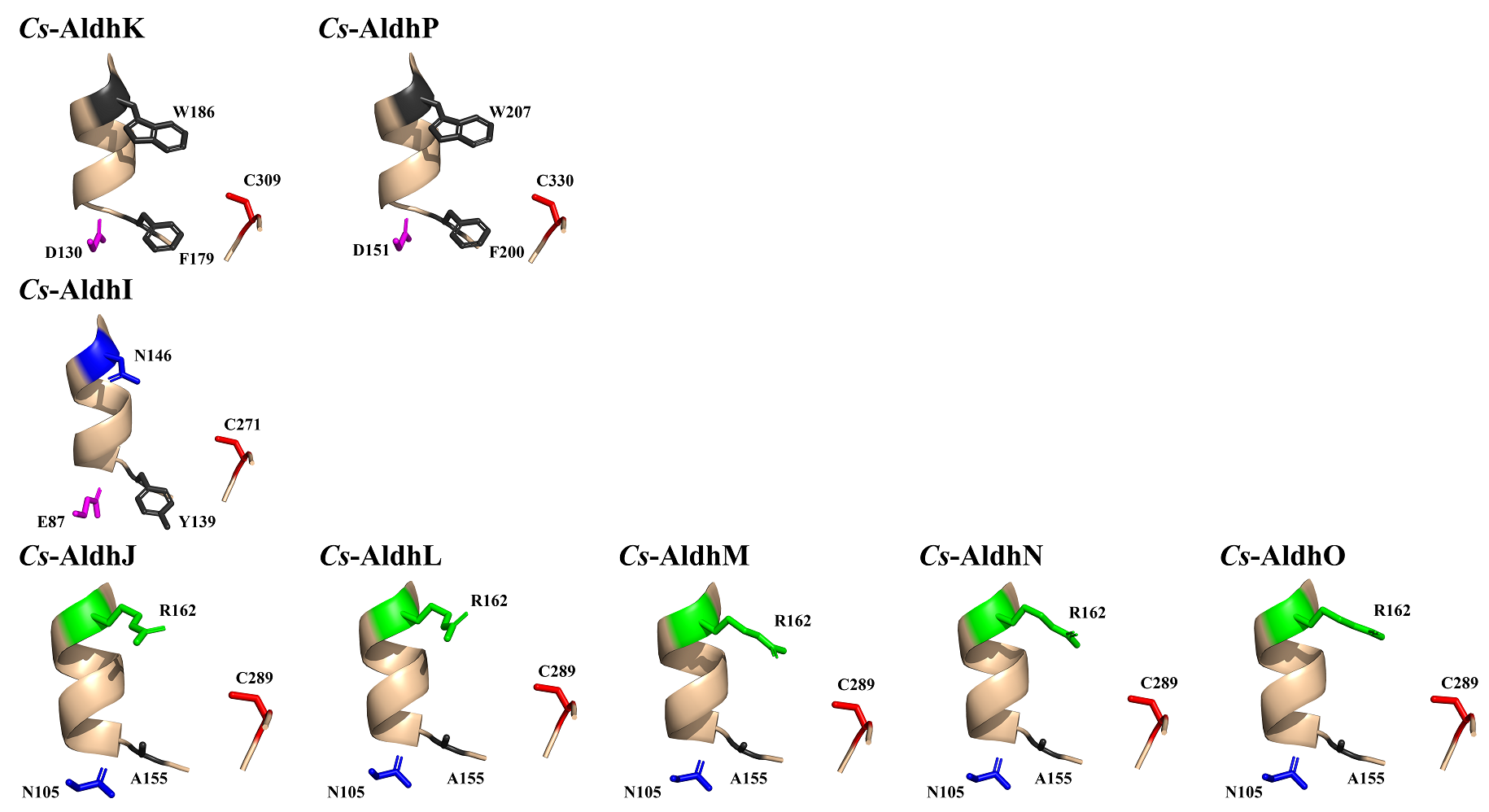


**Fig. S6. Predicted 3D models of *Cs*-Aldh substrate-binding channels generated by ColabFold and visualized in PyMOL.**

Five structural models were generated for each enzyme and aligned on the catalytic cysteine residue (C303) of *Cs*-AldhA for comparative analysis. The six horizontal rows correspond to the phylogenetic groups shown in Fig. 2. Each model highlights 13 amino acids (N170–K179 and V302–A304 in *Cs*-AldhA), with the backbone shown in ivory and four key side chains—D122, F171, W178, and C303—colored distinctly for clarity. Distances between three substrate-interacting residues and the catalytic cysteine (*v*–*z*) were measured in PyMOL and averaged across the five replicates (Table S4).

Residue types are color-coded as follows: positively charged (green), negatively charged (magenta), polar (blue), and nonpolar (black). The following reference points were used to define inter-residue distances:

– Arginine: center of the guanidino group

– Histidine: center of the imidazole ring

– Lysine: nitrogen atom of the *ε*-amino group

– Aspartic acid and glutamic acid: center of the carboxyl group

– Asparagine and glutamine: center of the amide group

– Alanine: center of the methyl group

– Phenylalanine, tryptophan, and tyrosine: center of the benzene ring

Residue numbering corresponds to *Cs*-AldhA and the aligned positions in each modeled enzyme (Fig. S3).

**(A) (B)**

**
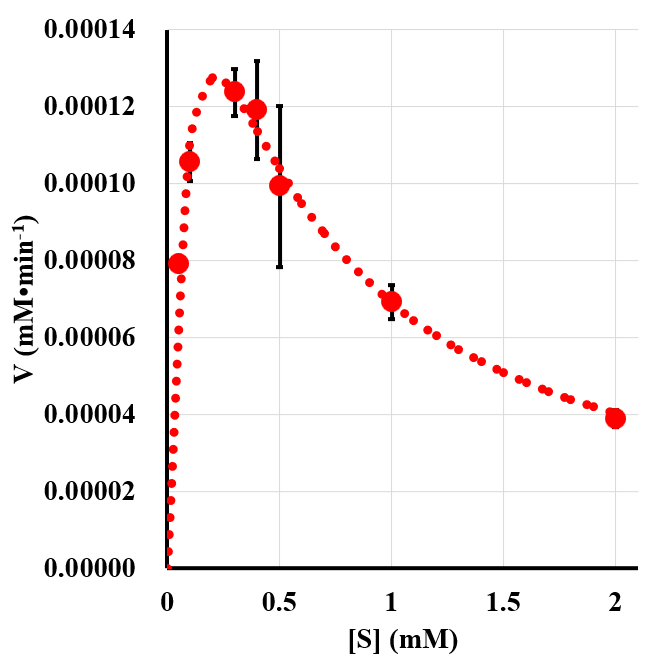

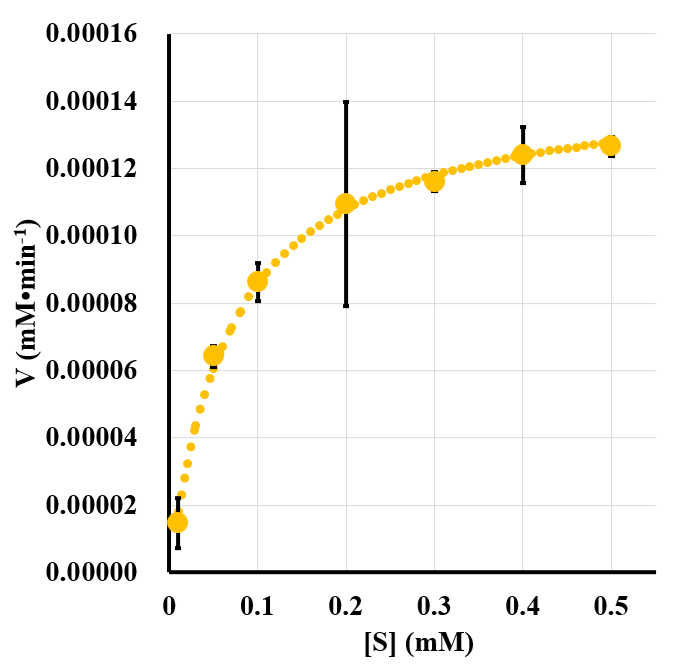
**

**Fig. S7. Kinetic analyses of *Cs*-AldhA and *Cs*-AldhK toward vanillin.**

Two *Cs*-Aldhs with low specific activities were further analyzed to determine their kinetic parameters.

**(A)** The catalytic behavior of *Cs*-AldhA followed the Haldane model, indicating substrate inhibition. The fitted equation was:

  V = (0.0002948 × [S]) / (0.1368 + [S] + [S]² / 0.3196)

  (R² = 0.9846)

The calculated kinetic parameters were: *Km* = 0.1368 mM, *Vmax* = 0.0002948 mM·min⁻¹, and *Ki* = 0.3196 mM.

**(B)** *Cs*-AldhK followed Michaelis–Menten kinetics. A Lineweaver–Burk plot yielded the following equation:

  1/V = 422.9238 / [S] + 7316.4983

  (R² = 0.9860)

From this, the kinetic parameters were calculated as *Km* = 57.8 µM and *Vmax* = 0.1367 µM·min⁻¹.

Abbreviations: [S], substrate concentration; V, reaction rate; *Km*, Michaelis constant; *Vmax*, maximum reaction rate; *Ki*, substrate inhibition constant; R², coefficient of determination indicating goodness-of-fit to the kinetic model.

**(A)** **(B)**


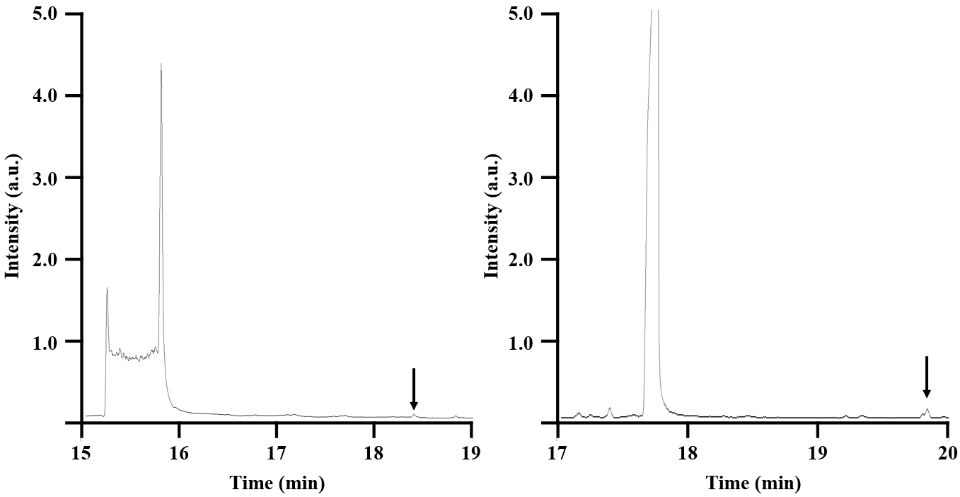

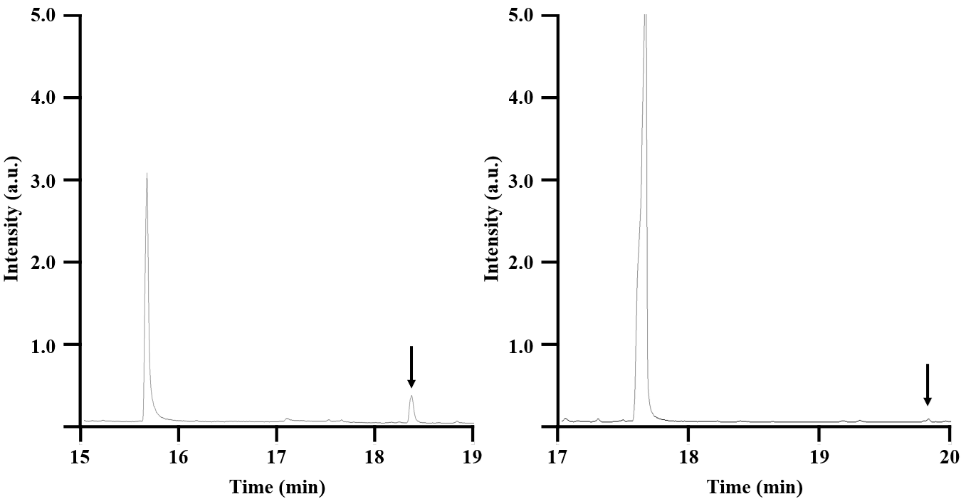


**(C)** **(D)**


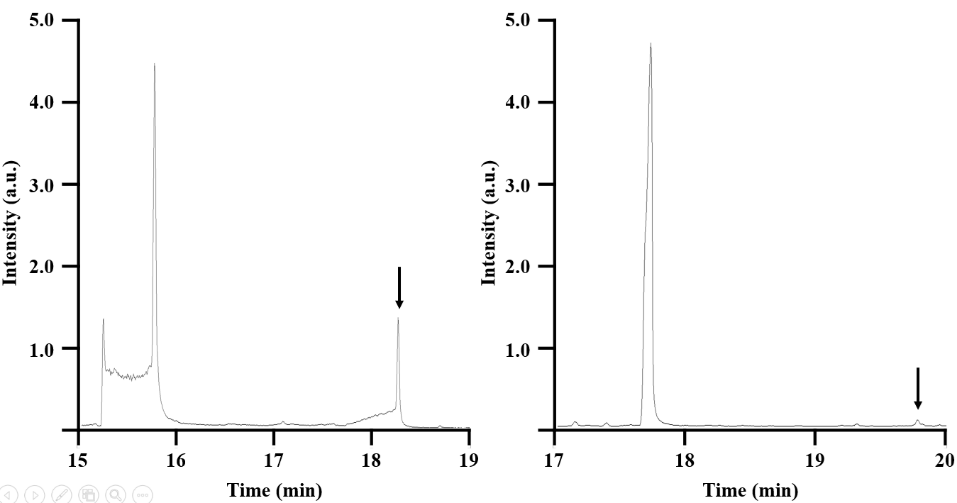

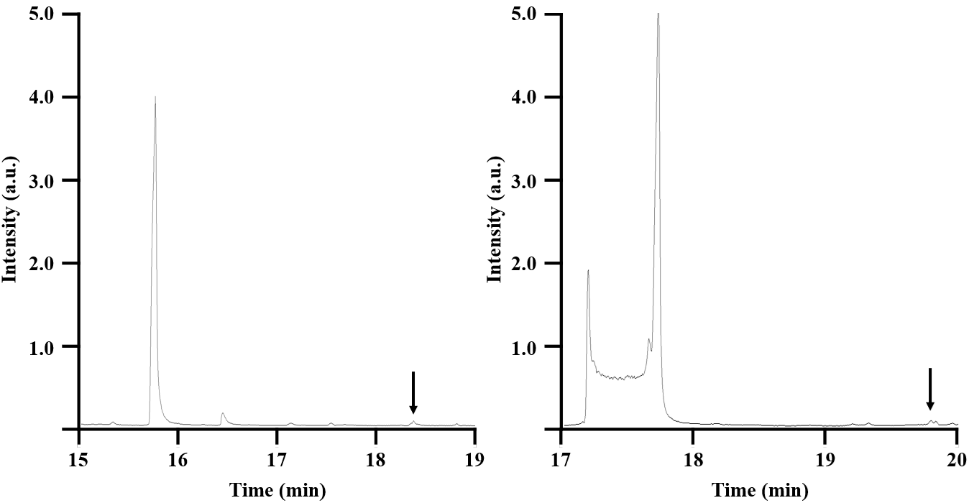


**(E)**  **(F)**


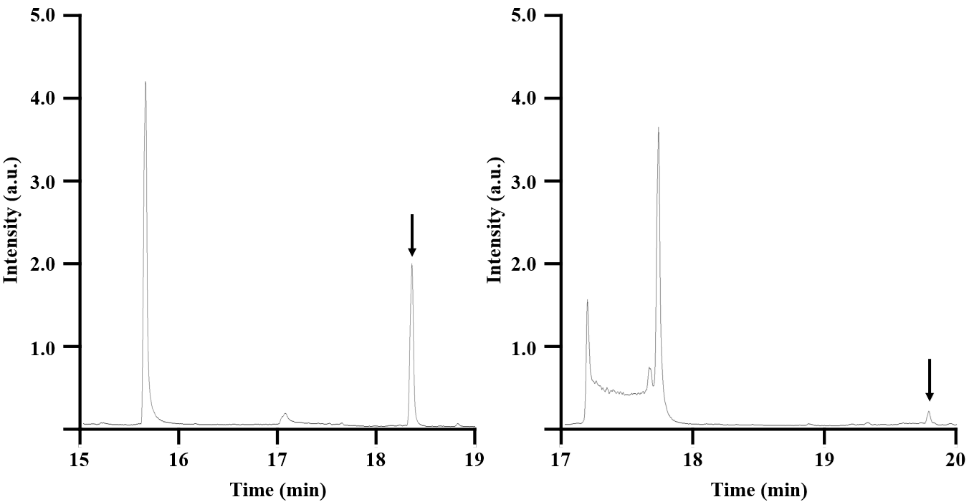

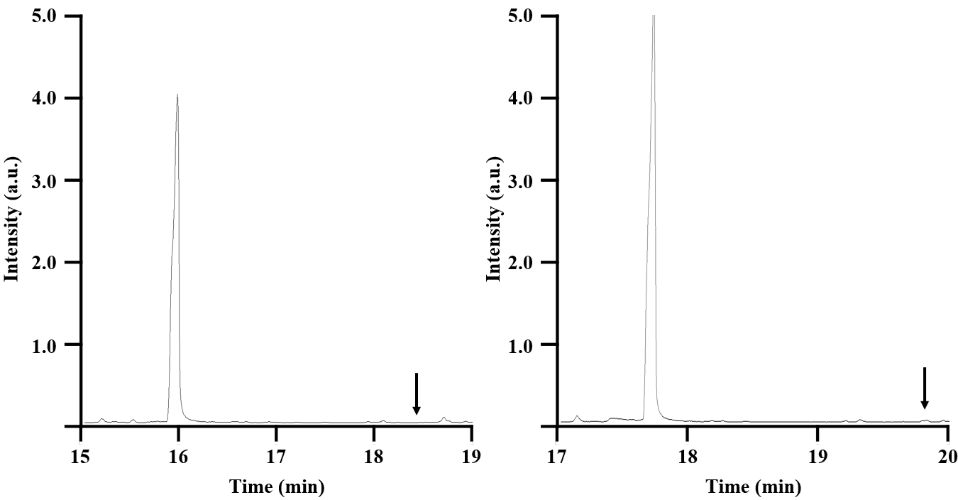


**(G)**  **(H)**


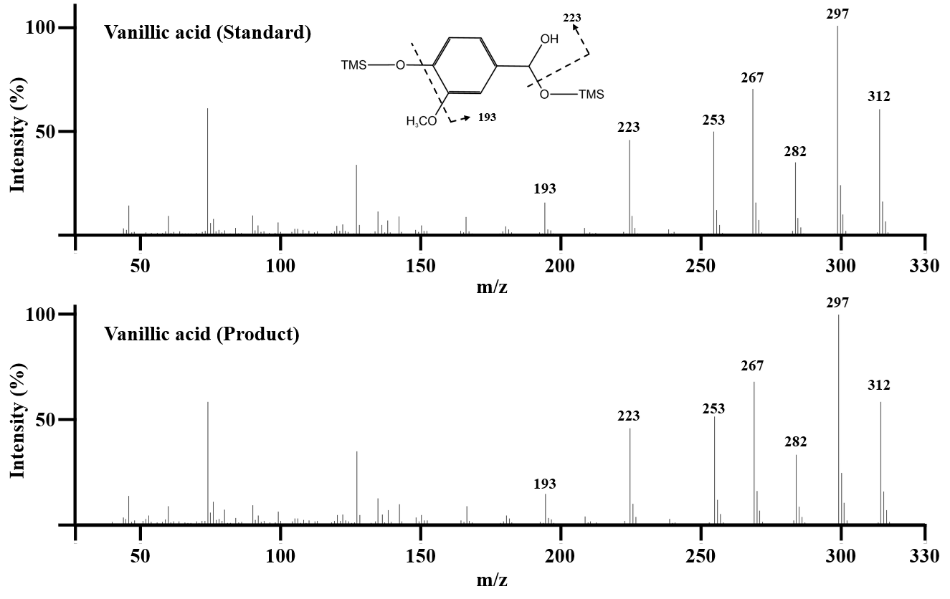

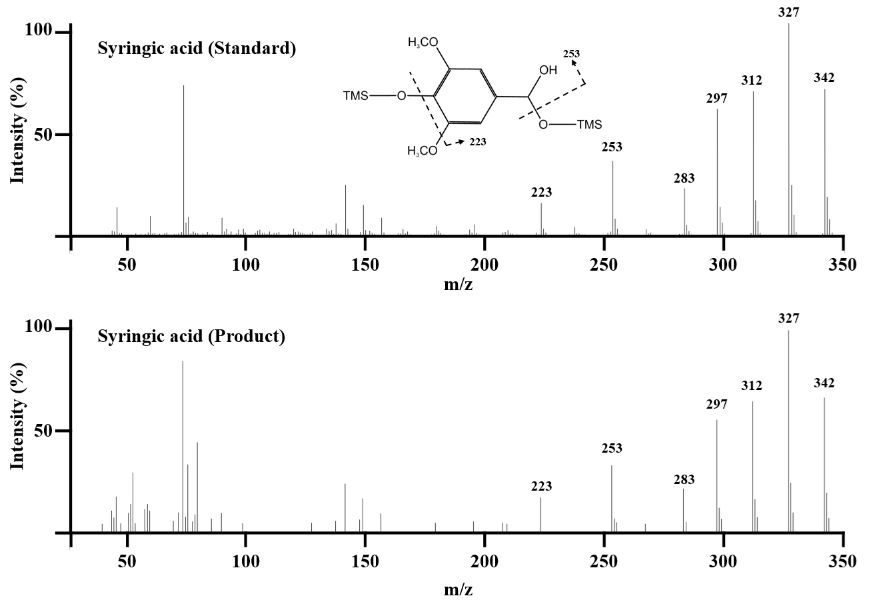


**Fig. S8. GC-MS analysis of enzymatic reaction mixtures of six *Cs*-Aldhs with vanillin and syringaldehyde.**

Each substrate (0.5 mM)—vanillin (left) or syringaldehyde (right)—was incubated with one of the following *Cs*-Aldhs: *Cs*-AldhA **(A)**, *Cs*-AldhE **(B)**, *Cs*-AldhH **(C)**, *Cs*-AldhI **(D)**, *Cs*-AldhJ **(E)**, and *Cs*-AldhK **(F)**. Reactions were carried out at 25°C for 2 h, followed by derivatization with TMS reagents and GC-MS analysis. Samples were analyzed using a DB-5MS column using helium as the carrier gas. The GC-MS system was operated in electron ionization (EI) mode at 70 eV, and mass spectra were acquired in full scan mode (*m/z* 50-600). Total ion chromatograms (TICs) are shown. The largest peaks on the left portion of each chromatogram **(A–F)** correspond to unreacted substrates—vanillin (15.79 min) and syringaldehyde (17.73 min). Black arrows indicate the formation of vanillic acid (18.38 min; left) and syringic acid (19.79 min; right). Panels G and H present the EI mass spectra of the TMS-derivatized standard and corresponding enzymatic products: vanillic acid-2TMS **(G)** and syringic acid-2TMS **(H)**. Major fragment ions were observed at *m/z* 297, 282, 267, and 253 for vanillic acid, and *m/z* 327, 312, 297, and 342 for syringic acid, consistent with sequential methyl group losses from the molecular ion (M⁺ −15, −30, −45). Additional fragments at *m/z* 223 and 193 (vanillic acid) and *m/z* 253 and 223 (syringic acid) are attributed to neutral loss of –O–TMS and –OCH₃ groups from hydroxyl and methoxyl moieties, respectively. These fragmentation patterns observed in enzymatic products were identical to those of the authentic standards, confirming their identities as vanillic acid and syringic acid.

**Table S1. Media compositions for fungal culture.**

1. **PDA plates**

**Component Amount Source**

potato dextrose agar 39 g Shimadzu Diagnostics Corporation, Tokyo, Japan

**Preparation:** Dissolved in 1 L of distilled water and autoclaved at 122 °C for 20 min.

**(B) BIII liquid medium**

**Component Amount Source**

(NH_4_)_2_SO_4_ 4.0 g Nacalai Tesque^*^

KH_2_PO_4_ 2.0 g FUJIFILM Wako^**^

MgSO_4_·7H_2_O 0.5 g Nacalai Tesque

CaCl_2_·2H_2_O 0.1 g Nacalai Tesque

**Preparation:** The solution was dissolved in 939 mL of distilled water, the pH was adjusted to 7.4, and the mixture was autoclaved at 122°C for 20 min.

**After cooling, the following components were added:**

50 mL of 20% (*w/v*) glucose solution (sterilized by 0.22 μm filtration)

10 mL of mineral elixir (see section C)

1 mL of thiamine-HCl solution (1 g/L; sterilized by 0.22 μm filtration)

**(C) Mineral elixir**

**Component Amount Source**

MgSO_4_·7H_2_O 3 g Nacalai Tesque

nitrilotriacetic acid 1.5 g DOJINDO Laboratories, Kumamoto, Japan

NaCl 1.0 g FUJIFILM Wako

MnSO_4_•5H_2_O 0.5 g FUJIFILM Wako

CoSO_4_·7H_2_O 0.1 g Nacalai Tesque

CaCl_2_·2H_2_O 0.1 g Nacalai Tesque

ZnSO_4_·7H_2_O 0.1 g Nacalai Tesque

FeSO_4_·H_2_O 0.1 g FUJIFILM Wako

CuSO_4_·5H_2_O 0.1 g FUJIFILM Wako

AlK(SO_4_)_2_·12H_2_O 0.01 g Nacalai Tesque

H_3_BO_3_ 0.01 g Nacalai Tesque

NaMoO_4_·2H_2_O 0.01 g Nacalai Tesque

**Preparation:** The solution was dissolved in 1 L of distilled water and sterilized by filtration through a 0.22 μm membrane filter.

^*^Nacalai Tesque, Inc., Kyoto, Japan

^**^FUJIFILM Wako Pure Chemical Corporation, Osaka, Japan

**Table S2. Primer sequences used in this study.**

Primer Names Sequences Purpose

*Cs*-*aldhA*.colony_F 5’-TTAAATACAGCTGCTCATCGTGACCTG-3’ *Cs*-*AldhA* colony PCR (forward)

*Cs*-*aldhA*.colony_R 5’-AGTACATCCTTCCAGTTGCACATTGAT-3’ *Cs*-*AldhA* colony PCR (reverse)

*Cs*-*aldhB*.colony_F 5’-TAAGTATAACCTTTCGCTAGTGTGTGG-3’ *Cs*-*AldhB* colony PCR (forward)

*Cs*-*aldhB*.colony_R 5’-CAGCCAGGCGCAAGTAAGTTATCGGTA-3’ *Cs*-*AldhB* colony PCR (reverse)

*Cs*-*aldhC*.colony_F 5’-GTATAGCCCTGGTGGTATGTGGGCTAT-3’ *Cs*-*AldhC* colony PCR (forward)

*Cs*-*aldhC*.colony_R 5’-AGCTCCGTGATTCCATTCATGCGCTAA-3’ *Cs*-*AldhC* colony PCR (reverse)

*Cs*-*aldhD*.colony_F 5’-TTATCGCTCCAAAGCATCTCCCATCGA-3’ *Cs*-*AldhD* colony PCR (forward)

*Cs*-*aldhD*.colony_R 5’-ACGCAACTATCGTACAGCTCTGGCTCT-3’ *Cs*-*AldhD* colony PCR (reverse)

*Cs*-*aldhE*.colony_F 5’-CGACTCGCGCTCACCTGGAGCGTAGCC-3’ *Cs*-*AldhE* colony PCR (forward)

*Cs*-*aldhE*.colony_R 5’-ATCGAGTGTTGCGGCACATACACCTTC-3’ *Cs*-*AldhE* colony PCR (reverse)

*Cs*-*aldhF*.colony_F 5’-CTGCAGCTTTTATAGCGAGGCCAAGTA-3’ *Cs*-*AldhF* colony PCR (forward)

*Cs*-*aldhF*.colony_R 5’-CTTAGAGATACTGCTACGCTAGGTCTT-3’ *Cs*-*AldhF* colony PCR (reverse)

*Cs*-*aldhG*.colony_F 5’-CCCCGAAGCCGGGCTCAGGCTGCCACT-3’ *Cs*-*AldhG* colony PCR (forward)

*Cs*-*aldhG*.colony_R 5’-GAGCGTCAAACAGTGGGGTCTTTGCCT-3’ *Cs*-*AldhG* colony PCR (reverse)

*Cs*-*aldhH*.colony_F 5’-GGGGGAGGCCTCCACTTCTAGCGGTTA-3’ *Cs*-*AldhH* colony PCR (forward)

*Cs*-*aldhH*.colony_R 5’-CTATCCTGTCTCTGCTCTGGTTCCATC-3’ *Cs*-*AldhH* colony PCR (reverse)

*Cs-aldhI.*colony_F 5’-CACGTGCAACACGGAAAGACGGAAGTA-3’ *Cs*-*AldhI* colony PCR (forward)

*Cs-aldhI*.colony_R 5’-ACCTAACCAACAGTGCGAATGGTCATA-3’ *Cs*-*AldhI* colony PCR (reverse)

*Cs-aldhJ*.colony_F 5’-CATTCTTGGCGTCTTGAGCAGTACATA-3’ *Cs*-*AldhJ* colony PCR (forward)

*Cs-aldhJ*.colony_R 5’-TTGCTTTTCGGACAGGTATATAGTACA-3’ *Cs*-*AldhJ* colony PCR (reverse)

*Cs-aldhK*.colony_F 5’-GGCGTCGCGCGCGTTCTCACATTCCTG-3’ *Cs*-*AldhK* colony PCR (forward)

*Cs-aldhK*.colony_R 5’-ATGAAATCCAATGTCAATTCCAAGCAT-3’ *Cs*-*AldhK* colony PCR (reverse)

*Cs-aldhL*.colony_F 5’-GCAGCAGAGTCAATTCTCAGGCCCGCA-3’ *Cs*-*AldhL* colony PCR (forward)

*Cs-aldhL*.colony_R 5’-GAATTCTGCTCGTATCACGCACCGAAT-3’ *Cs*-*AldhL* colony PCR (reverse)

*Cs-aldhM*.colony_F 5’-GAATAGCGCCCCCACTCACTTAAAGGA-3’ *Cs*-*AldhM* colony PCR (forward)

*Cs-aldhM*.colony_R 5’-GCCAGTGGCACCGCTGCACAGGAAAAT-3’ *Cs*-*AldhM* colony PCR (reverse)

*Cs-aldhN*.colony_F 5’-GACGAGCTGATTCTCTGTTGCATTGCG-3’ *Cs*-*AldhN* colony PCR (forward)

*Cs-aldhN*.colony_R 5’-ACATGCATATGATCCAATACATTCCAC-3’ *Cs*-*AldhN* colony PCR (reverse)

*Cs-aldhO*.colony_F 5’-AGCTGCACGCCTTTAGTTTGCGATGAG-3’ *Cs*-*AldhO* colony PCR (forward)

*Cs-aldhO*.colony_R 5’-ATGTCAAAACGCCCATATCCGGTGGCA-3’ *Cs*-*AldhO* colony PCR (reverse)

*Cs-aldhP*.colony_F 5’-GGATGCGGGGCTCCAGCCTACCCCGTG-3’ *Cs*-*AldhP* colony PCR (forward)

*Cs-aldhP*.colony_R 5’-GACATCAAGATCAGGCTAGAATTACAT-3’ *Cs*-*AldhP* colony PCR (reverse)

*Cs-aldhA*.cds_F 5’-CATATGCCTGAGACCTTCGTGTACCAG-3’ *Cs-AldhA* CDS cloning (forward)

*Cs-aldhA*.cds_R 5’-CATATGTCAATGGTGATGGTGATGGTGTGCGAGGTTGACCTGGACCGCCTTG-3’ *C*s-*AldhA* CDS cloning (reverse)

*Cs-aldhB*.cds_F 5’-CATATGCCGTCAACTTTCGACTACACC-3’ *Cs-AldhB* CDS cloning (forward)

*Cs-aldhB*.cds_R 5’-CATATGTCAATGGTGATGGTGATGGTGGGCGATATTGATCTGGATCGCCTTAAC-3’ *C*s-*AldhB* CDS cloning (reverse)

*Cs-aldhC*.cds_F 5’-CATATGCCATCGACTTTCGAGTATACC-3’ *Cs-AldhC* CDS cloning (forward)

*Cs-aldhC*.cds_R 5’-CATATGTCAATGGTGATGGTGATGGTGAGCAAGGTTGATTTGAATCGCCTTG-3’ *C*s-*AldhC* CDS cloning (reverse)

*Cs-aldhD*.cds_F 5’-CATATGGCCCAGCCTGTTGAGATCTC-3’ *Cs-AldhD* CDS cloning (forward)

*Cs-aldhD*.cds_R 5’-CATATGTCAATGGTGATGGTGATGGTGGAAAGGCCAGTCGAGTTTCTCCCCGAAG-3’ *C*s-*AldhD* CDS cloning (reverse)

*Cs-aldhE*.cds_F 5’-CATATGGCCAGCACGGCGGCAGCAAAC-3’ *Cs-AldhE* CDS cloning (forward)

*Cs-aldhE*.cds_R 5’-CATATGTCAATGGTGATGGTGATGGTGGAGTGGCCACAGACCGCCGATGG-3’ *C*s-*AldhE* CDS cloning (reverse)

*Cs-aldhF*.cds_F 5’-CATATGGGTACTACTTTGGCTAACGGC-3’ *Cs-AldhF* CDS cloning (forward)

*Cs-aldhF*.cds_R 5’-CATATGTCAATGGTGATGGTGATGGTGAGCGTGAACTGTGATGGTCTGAATC-3’ *C*s-*AldhF* CDS cloning (reverse)

*Cs-aldhF*.cdsN_R 5’-CATATGTCAAGCGTGAACTGTGATGGTCTGAATC-3’ *C*s-*AldhF* CDS cloning (reverse, N-His)

*Cs-aldhG*.cds_F 5’-CATATGGACACTCAGGAGGAAATCCTCTC-3’ *Cs-AldhG* CDS cloning (forward)

*Cs-aldhG*.cds_R 5’-CATATGTCAATGGTGATGGTGATGGTGTCCGTAGCGTTTCTCCGCGCTATCGTC-3’ *C*s-*AldhG* CDS cloning (reverse)

*Cs-aldhH*.cds_F 5’-CATATGATGTCTTTCTCTAATGATTTTCTC-3’ *Cs-AldhH* CDS cloning (forward)

*Cs-aldhH*.cds_R 5’-CATATGTCAATGGTGATGGTGATGGTGGGCATGGATCGTCATGGTCTGTGTG-3’ *C*s-*AldhH* CDS cloning (reverse)

*Cs-aldhI*.cds_F 5’-CATATGGCTGTTCCGGAGACGCAGACAAC-3’ *Cs-AldhI* CDS cloning (forward)

*Cs-aldhI*.cds_R 5’-CATATGTCAATGGTGATGGTGATGGTGCGAAGTCTTAATCTTCATGTGCACAG-3’ *C*s-*AldhI* CDS cloning (reverse)

*Cs-aldhJ*.cds_F 5’-CATATGTCTGTCCCATTCACATCACTG-3’ *Cs-AldhJ* CDS cloning (forward)

*Cs-aldhJ*.cds_R 5’-CATATGTCAATGGTGATGGTGATGGTGTCCCACAACCGGATACTGTTGGTTCTG-3’ *C*s-*AldhJ* CDS cloning (reverse)

*Cs-aldhK*.cds_F 5’-CATATGTCTGCACCGACGAGCAGTCTG-3’ *Cs-AldhK* CDS cloning (forward)

*Cs-aldhK*.cds_R 5’-CATATGTCAATGGTGATGGTGATGGTGGCGATGGGTTGGCATGTTCACAGATG-3’ *C*s-*AldhK* CDS cloning (reverse)

*Cs-aldhL*.cds_F 5’-CATATGAGCATCCCTTTCACGTCTCTC-3’ *Cs-AldhL* CDS cloning (forward)

*Cs-aldhL*.cds_R 5’-CATATGTCAATGGTGATGGTGATGGTGACCAACAACCGGGTACCACGGATTCTG-3’ *C*s-*AldhL* CDS cloning (reverse)

*Cs-aldhM*.cds_F 5’-CATATGTCCGTGCCTTTCACCCAACTC-3’ *Cs-AldhM* CDS cloning (forward)

*Cs-aldhM*.cds_R 5’-CATATGTCAATGGTGATGGTGATGGTGCCCTAATCCACCAATACCGTCAGCAG-3’ *C*s-*AldhM* CDS cloning (reverse)

*Cs-aldhM*.cdsN_R 5’-CATATGTCACCCTAATCCACCAATACCGTCAGCAG-3’ *C*s-*AldhM* CDS cloning (reverse, N-His)

*Cs-aldhN*.cds_F 5’-CATATGTCTGTCCCACTCACATCACTC-3’ *Cs-AldhN* CDS cloning (forward)

*Cs-aldhN*.cds_R 5’-CATATGTCAATGGTGATGGTGATGGTGTCCAACAAGAGGGTACGGTACCGTATAG-3’ *C*s-*AldhN* CDS cloning (reverse)

*Cs-aldhO*.cds_F 5’-CATATGTCCGTCCCTTTCACGTCGCTC-3’ *Cs-AldhO* CDS cloning (forward)

*Cs-aldhO*.cds_R 5’-CATATGTCAATGGTGATGGTGATGGTGCCCCAAGCCTCCAAGGCCGTCTC-3’ *C*s-*AldhO* CDS cloning (reverse)

*Cs-aldhO*.cdsN_R 5’-CATATGTCACCCCAAGCCTCCAAGGCCGTCTC-3’ *C*s-*AldhO* CDS cloning (reverse, N-His)

*Cs-aldhP*.cds_F 5’-CATATGTCGTCCAATTTATCTCGAACG-3’ *Cs-AldhP* CDS cloning (forward)

*Cs-aldhP*.cds_R 5’-CATATGTCAATGGTGATGGTGATGGTGGTTGTGCGTAGGCATATCCACAGATG-3’ *C*s-*AldhP* CDS cloning (reverse)

The *Nde*I sites are underlined. Six histidine codons are highlighted in gray. The term “colony” in the primer name column indicates that these primers were designed based on the *C. subvermispora* strain B genome available on the JGI homepage (http://genome.jgi-psf.org/Cersu1/Cersu1.home.html) and were used to confirm the presence of the corresponding Aldh genes in *C. subvermispora* ATCC 90467. The term “cds” in the primer name column indicates that these primers were designed based on the terminal coding sequences of Aldh from *C. subvermispora* ATCC 90467 and were used to amplify the full coding sequences of the corresponding genes for sequencing and transformation. N-His indicates the addition of a His-tag at the N-terminus; therefore, the reverse primer does not include six histidine codons.

**Table S3. Transmembrane regions of *Cs*-Aldhs predicted by the SOSUI and TMHMM algorithms.**

Protein Algorithms Region Sequence

*Cs*-AldhF

SOSUI ver. 1.11* 149 – 171 (23 aa) GVCAAITPFNFPLSLVAWKLSPA

TMHMM ver. 2.0** 142 – 163 (22 aa) VVRYVPVGVCAAITPFNFPLSL (very low possibility, 8.7 %)

*Cs*-AldhH

SOSUI ver. 1.11* 141 – 163 (23 aa) VCAAITPFNFPLSLMVWKLAPAL

TMHMM ver. 2.0** 133 – 155 (23 aa) IVRYVPVGVCAAITPFNFPLSLM (very low possibility, 2.8 %)

*Cs*-AldhI

SOSUI ver. 1.11* No transmembrane region

TMHMM ver. 2.0** 126 – 152 (27 aa) VPLGVVLVVAPWNYPYLVSVNSVLPAL (low possibility, 21.0 %)

*Cs*-AldhJ

SOSUI ver. 1.11* 94 – 116 (23 aa) ETAAVDFVLLFNIMATIGIAREY

150 – 172 (23 aa) IAPWNAPVVLSLRAMAIPILCGN

TMHMM ver. 2.0** No transmembrane region

*Cs*-AldhK

SOSUI ver. 1.11* No transmembrane region

TMHMM ver. 2.0** 166 – 189 (24 aa) LPLGVCASIAPFNFPAMIPLWTLP (low possibility, 11.1 %)

*Cs*-AldhM

SOSUI ver. 1.11 144 – 166 (23 aa) KGVVFAIAPWNAPLVLTIRAIGT

TMHMM ver. 2.0 101 – 116 (16 aa) LSGFNLVVAVEFLRLY (very low possibility, 1.1 %)

146 – 166 (21 aa) VVFAIAPWNAPLVLTIRAIGT (very low possibility, 2.0 %)

*Cs*-AldhP

SOSUI ver. 1.11* No transmembrane region

TMHMM ver. 2.0** 187 – 210 (24 aa) LPLGVCASIAPFNFPAMIPLWTIP (low possibility, 27.0 %)

Prior to the expression of *Cs*-Aldhs, the existence of transmembrane regions was investigated by querying the complete amino acid sequences using the SOSUI ver. 1.11 (SOSUI) and TMHMM ver. 2.0 (TMHMM) algorithms. The unlisted *Cs*-*aldh*s did not contain any predicted transmembrane regions.

*SOSUI v. 1.11 (https://harrier.nagahama-i-bio.ac.jp/sosui/)

**TMHMM v.2.0 (http://www.cbs.dtu.dk/services/TMHMM/)

**Table S4. Calculated distances between side chains of each *Cs*-Aldh.**

Protein *u v w x y z*

*Cs*-AldhA 5.28 ± 0.075 10.18 ± 0.13 7.94 ± 0.14 7.08 ± 0.15 6.80 ± 0.13 11.90 ± 0.14

*Cs*-AldhB 5.36 ± 0.080 10.00 ± 0.14 8.14 ± 0.10 6.70 ± 0.11 7.56 ± 0.10 11.70 ± 0.14

*Cs*-AldhC 5.18 ± 0.12 10.10 ± 0.14 8.30 ± 0.063 6.86 ± 0.14 7.64 ± 0.10 11.74 ± 0.21

*Cs*-AldhD 5.36 ± 0.049 10.20 ± 0.063 8.14 ± 0.080 6.88 ± 0.16 7.48 ± 0.15 11.96 ± 0.14

*Cs*-AldhE 4.90 ± 0.089 9.84 ± 0.080 7.60 ± 0.11 6.88 ± 0.19 6.44 ± 0.14 11.32 ± 0.13

Cs-AldhG 4.38 ± 0.098 9.06 ± 0.76 8.20 ± 0.54 6.86 ± 0.10 6.70 ± 0.51 10.56 ± 0.049

*Cs*-AldhF 4.98 ± 0.075 10.20 ± 0.14 8.56 ± 0.14 7.72 ± 0.075 7.26 ± 0.080 11.80 ± 0.063

*Cs*-AldhH 4.98 ± 0.040 10.34 ± 0.39 8.62 ± 0.098 7.62 ± 0.075 7.28 ± 0.098 11.72 ± 0.075

*Cs*-AldhK 5.22 ± 0.040 9.38 ± 0.19 7.56 ± 0.19 5.82 ± 0.28 6.64 ± 0.19 10.68 ± 0.19

Cs-AldhP 5.22 ± 0.098 9.88 ± 0.19 7.98 ± 0.23 5.70 ± 0.34 6.84 ± 0.17 10.56 ± 0.25

*Cs*-AldhI 5.30 ± 0.089 10.92 ± 0.15 10.70 ± 0.063 7.16 ± 0.22 9.42 ± 0.17 11.38 ± 0.20

*Cs*-AldhJ 4.10 ± 0.14 10.78 ± 0.30 7.82 ± 0.28 8.12 ± 0.28 7.16 ± 0.22 11.84 ± 0.24

*Cs*-AldhL 4.62 ± 0.075 11.06 ± 0.48 7.58 ± 0.61 8.50 ± 0.79 7.02 ± 0.26 13.02 ± 0.82

*Cs*-AldhM 4.10 ± 0.13 11.16 ± 0.62 7.90 ± 0.47 8.10 ± 0.14 6.50 ± 0.24 11.76 ± 0.080

*Cs*-AldhN 4.32 ± 0.098 11.32 ± 0.41 7.98 ± 0.23 8.02 ± 0.098 6.64 ± 0.12 12.00 ± 0.063

*Cs*-AldhO 4.26 ± 0.049 11.38 ± 0.32 8.12 ± 0.15 8.20 ± 0.15 7.02 ± 0.31 12.20 ± 0.11

Reference distances are shown in Fig. S3 (highlighted in the box). Each distance is defined as follows for *Cs*-AldhA: *u* represents the distance from D122 to F171, *v* from D122 to W178, *w* from F171 to W178, *x* from F171 to C303, *y* from W178 to C303, and *z* from D122 to C303. The unit is Å. Calculations were performed five times.
